# Transcriptionally defined amygdala subpopulations play distinct roles in innate social behaviors

**DOI:** 10.1038/s41593-023-01475-5

**Published:** 2023-11-09

**Authors:** Julieta E. Lischinsky, Luping Yin, Chenxi Shi, Nandkishore Prakash, Jared Burke, Govind Shekaran, Maria Grba, Joshua G. Corbin, Dayu Lin

**Affiliations:** 1 Neuroscience Institute, New York University School of Medicine, New York, NY USA; 2 Hunter College, New York, NY USA; 3 Center for Neuroscience Research, Children’s National Hospital, Washington, DC USA; 4 Center for Neural Science, New York University, New York, NY USA; 5 Department of Psychiatry, New York University School of Medicine, New York, NY USA

**Keywords:** Social behaviour, Sensory processing, Neural circuits, Cellular neuroscience

## Abstract

Social behaviors are innate and supported by dedicated neural circuits, but the molecular identities of these circuits and how they are established developmentally and shaped by experience remain unclear. Here we show that medial amygdala (MeA) cells originating from two embryonically parcellated developmental lineages have distinct response patterns and functions in social behavior in male mice. MeA cells expressing the transcription factor Foxp2 (MeA^Foxp2^) are specialized for processing male conspecific cues and are essential for adult inter-male aggression. By contrast, MeA cells derived from the *Dbx1* lineage (MeA^Dbx1^) respond broadly to social cues, respond strongly during ejaculation and are not essential for male aggression. Furthermore, MeA^Foxp2^ and MeA^Dbx1^ cells show differential anatomical and functional connectivity. Altogether, our results suggest a developmentally hardwired aggression circuit at the MeA level and a lineage-based circuit organization by which a cell’s embryonic transcription factor profile determines its social information representation and behavioral relevance during adulthood.

## Abstract (web summary)

The authors describe the connectivity, response profile and behavioral roles of two transcriptionally defined amygdala populations from separate embryonic lineages and show how responses of one population change with social experience.

## Main

Innate social behaviors, such as mating, fighting and parenting, are indispensable for the survival and propagation of a species and, therefore, are present widely in the animal kingdom. These behaviors are considered innate as they can take place without learning, although the efficiency in performing these behaviors can be improved with repeated execution^1^. The developmental mechanisms for the establishment of innate social behaviors and the role of experience in shaping these circuits remain poorly understood.

An array of interconnected brain regions, collectively called the social behavior network (SBN), was proposed to be important for diverse social behaviors^2,3^. The medial amygdala (MeA), especially its posterior division (MeAp), is considered a key node of the SBN based on its connectivity, activity, gonadal hormone receptor expression and numerous lesion studies^2^. MeA is the primary recipient of accessory olfactory bulb (AOB) inputs, the exclusive relay of the vomeronasal organ (VNO) specialized in detecting pheromones^4^. Volatile information also reaches MeA cells via the cortical amygdala^5,6^. Consistent with the strong olfactory inputs, immediate early gene mapping, in vivo electrophysiological recordings and Ca^*2+*^ imaging have all revealed increased MeA activity after exposure to conspecific and heterospecific chemosensory cues^6–10^. Unsurprisingly, MeA lesion causes deficits in multiple social behaviors, including male sexual behavior, aggression and maternal behaviors^11–15^. These studies collectively support an important role for the MeA in processing and relaying olfactory information related to conspecifics.

Recent functional experiments suggest a more direct role of the MeAp in driving social behaviors. Hong et al.^16^ first showed that optogenetic activation of GABAergic MeAp cells acutely induced mounting or attack in male mice depending on stimulation intensity. Later, Unger et al.^17^ reported that silencing or ablating aromatase-expressing MeAp cells decreased aggression in both sexes. Work from three recent studies^18–20^ found that activation of the projection from MeA^Npy1r^, MeA^D1R^ or MeA^CaMKII^ cells to the bed nucleus of the stria terminalis (BNST) promoted male aggression. Beyond aggression, MeA GABAergic cells were also found to drive pup grooming, infanticide and allogrooming^21,22^.

These results raised several questions regarding the MeA function in social behaviors. First, are there dedicated MeA subpopulations for distinct social behaviors, or can any random subsets of MeA cells generate any social behavior in a context-dependent and intensity-dependent manner? An answer to this question remains unclear as activating multiple subpopulations of MeA cells can all induce aggression^17–20^, whereas activating the same GABAergic MeA population induces diverse social behaviors^16,21,22^. Second, how much of the MeA cell response is developmentally hardwired versus determined by adult experience? Choi et al.^7^ found that MeA cells relevant for social behaviors and predator defense are marked by different members of the Lhx family of transcription-factor-encoding genes, suggesting developmental hardwiring of social versus non-social signals. However, recent imaging studies revealed that MeA cell responses to social stimuli could be altered with adult experience, suggesting that the exact social response of MeA cells may not be pre-determined^10^.

**Fig. 1:**
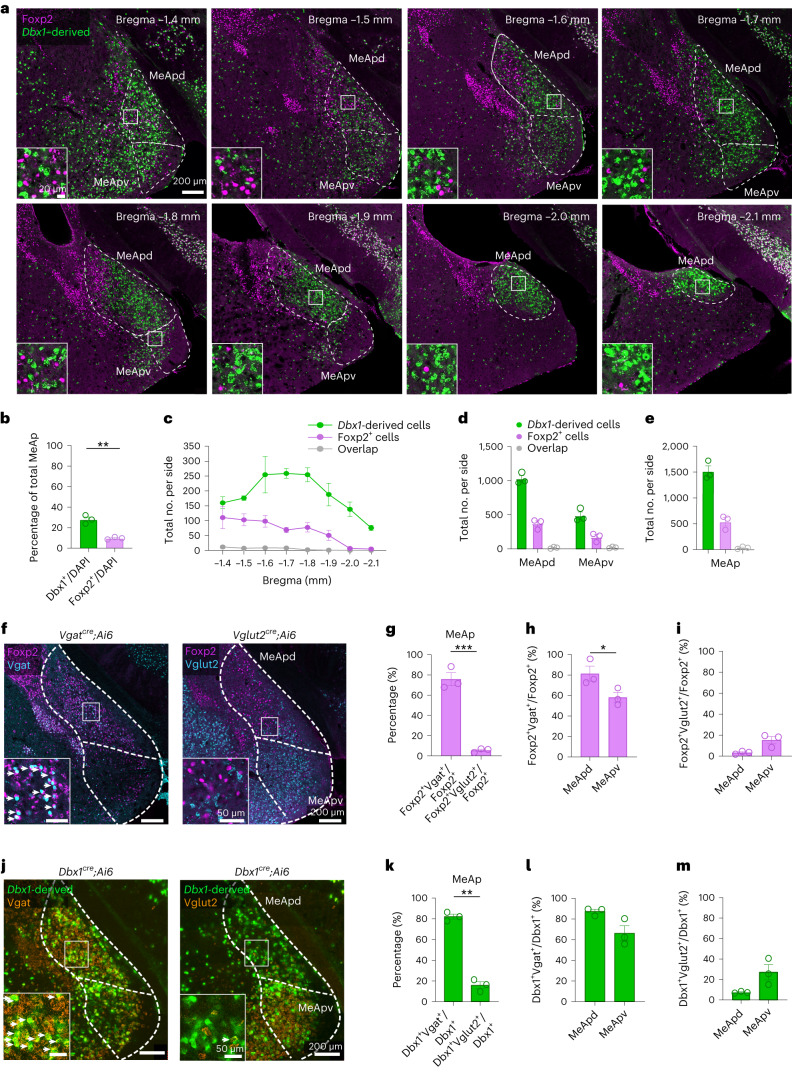
MeA^Foxp2^ and MeA^Dbx1^ cells are essentially non-overlapping transcriptionally defined subpopulations. **a**, Immunostaining of Foxp2 and GFP (*Dbx1*-derived cells) in the MeAp of *Dbx1*^*cre*^*;Ai6* male mice. Left bottom shows the enlarged view of boxed areas. **b**, Percentage of MeA^Foxp2^ and MeA^Dbx1^ cells in the total MeAp population. **c,** The number of Foxp2-only, *Dbx1*-derived-only and double-positive cells in each side of the MeAp from bregma −1.4 mm to −2.1 mm. **d**, The total number of counted Foxp2-only, *Dbx1*-derived-only and double-positive cells in each side of the posterodorsal and posteroventral MeA (MeApd and MeApv). **e**, The total number of Foxp2-only, *Dbx1*-derived-only and double-positive cells in each side of the MeAp. **f**, Immunostaining of Foxp2 (magenta) and GFP (marking Vgat or Vglut2, cyan) in the MeA of *Vgat*^*cre*^*;Ai6* or *Vglut2*^*cre*^*;Ai6* male mice. The left bottom shows the enlarged view of boxed areas. **g**, Percentage of MeA^Foxp2^ cells overlapping with Vgat^+^ or Vglut2^+^ cells in the MeAp. **h**, Percentage of Foxp2^+^Vgat^+^ cells over the total Foxp2^+^ cells in the MeApd and MeApv. **i**, Percentage of Foxp2^+^Vglut2^+^ cells over the total Foxp2^+^ cells in the MeApd and MeApv. **j**, Triple in situ hybridization of Vgat (left, orange), Vglut2 (right, orange) and GFP (marking *Dbx1*-derived cells, green) in the MeAp of *Dbx1*^*cre*^*;Ai6* male mice. Left bottom shows the enlarged view of boxed areas. **k**, Percentage of MeA^Dbx1^ cells overlapping with Vgat^+^ or Vglut2^+^ cells in the MeAp. **l**, Percentage of *Dbx1-*derived^+^Vgat^+^ cells over the total *Dbx1-*derived cells in the MeApd and MeApv. **m**, Percentage of *Dbx1-*derived^+^Vglut2^+^ cells over the total *Dbx1-*derived cells in the MeApd and MeApv. For **b**–**e** and **g**–**i**, every third of 50-µm brain sections was counted. For **k**–**m**, every sixth of 20-µm brain sections was counted. The Allen Brain Reference Atlas was used to determine the MeAp subdivisions. *n*, number of animals*. n* = 3 mice for all groups. **b**, Unpaired *t*-test, *P* = 0.0018. **g**–**i**,**k**–**m**, Paired *t*-tests; *P* = 0.0004 (**g**), *P* = 0.0132 (**h**), *P* = 0.0075 (**k**). All statistical tests are two-tailed. Data are mean ± s.e.m. *P < 0.05, **P < 0.01, ***P < 0.001, otherwise *P* > 0.05. See Source Data Fig. 1 for more detailed statistics. Source data

Taken together, despite being a central node of the SBN, how the MeA mediates social behaviors remains elusive. We previously identified two distinct MeA populations that arise from separate embryonic lineages in the telencephalic preoptic area (POA), marked by the transcription factors Dbx1 and Foxp2 (refs. ^9,23^). In adults, although Dbx1 is no longer expressed in the MeA, *Dbx1* lineage cells remain distinct from Foxp2-expressing cells despite being spatially intermingled^9^ (Fig. 1). Addtionally, these two subpopulations differ in their gene expression patterns and intrinsic electrophysiological properties^9^. Therefore, we reason that these two developmentally distinct and transcriptionally defined subpopulations could provide a unique opportunity to address whether social cue representation and social function of MeA cells are predetermined by their developmental lineage. In the present study, we compared the neuronal responses, functions and connectivity of MeA^Dbx1^ and MeA^Foxp2^ cells in male social behaviors and revealed the response pattern of MeA^Foxp2^ cells over development. Our findings highlight the interplay between nature (development) versus nurture (experience) in shaping social sensory representations, supporting a framework by which neuronal function and circuitry are developmentally defined.

## Results

### Distribution of MeA^Dbx1^ and MeA^Foxp2^ cells in male mice

**Extended Data Fig. 1:**
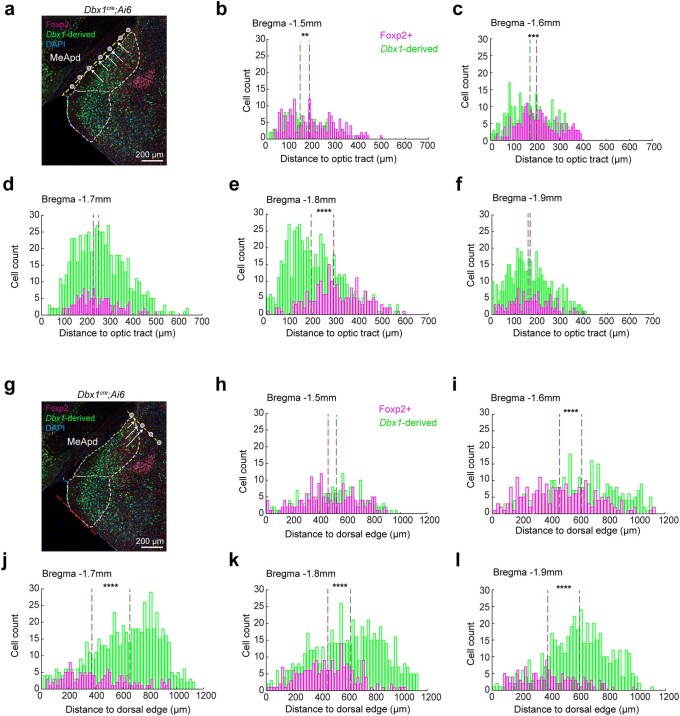
Characterization of MeA^Foxp2^ and MeA^Dbx1^ cell distribution in the MeApd, related to Fig. 1. **(a)** Representation of a histological MeAp image. The open circles and the dashed line indicate the optic tract used to determine the medial/lateral location of MeApd^Foxp2^ and MeApd^Dbx1^ cells. **(b-f)** Distribution of MeApd^Foxp2^ (magenta) and MeApd^Dbx1^ (green) cell distance to the optic tract at Bregma −1.5 mm **(b)**, −1.6 mm **(c)**, −1.7 mm **(d)**, −1.8 mm **(e)** and −1.9 mm **(f)**. **(g)** Representation of a histological MeAp image. The open circles and the dashed line indicate the MeA dorsal edge used to determine the dorsal/ventral location of MeApd^Foxp2^ and MeApd^Dbx1^ cells. **(h-l)** Distribution of MeA^Foxp2^ (magenta) and MeA^Dbx1^ (green) cell distance from the dorsal edge at Bregma −1.5 mm **(h)**, −1.6 mm **(i)**, −1.7 mm **(j)**, −1.8 mm **(k)** and −1.9 mm **(l)**. Magenta and green dashed lines denote the median medial-lateral **(b-f)** and ventral-lateral **(h-l)** locations of the MeApd^Foxp2^ and MeApd^Dbx1^ cells, respectively. White arrows indicate how the distance to the optic tract **(a)** or the dorsal edge of the MeApd **(g)** is calculated. *n* = *133-568* from 3 animals for each group (where *n* is the number used for statistical analysis). **(b-f, h-l)** Two-tailed Mann Whitney tests; **(b)** p = 0.0018, **(c)** p = 0.0002, **(e, i-l)** p < 0.0001. **p < 0.01; ***p < 0.001; ***p < 0.0001. Otherwise, p > 0.05. See Source Data Extended Data Fig. 1 for more detailed statistics. Source data

To visualize the spatial distribution of MeA^Dbx1^ and MeA^Foxp2^ cells in adults, we crossed *Dbx1*^*cre*^ mice^24^ with a ZsGreen reporter line (Ai6)^25^ and immunostained for Foxp2. MeA^Dbx1^ cells make up approximately 28% of total posterior MeA cells (MeAp, bregma level −1.4 mm to −2.1 mm) and are found in both dorsal and ventral subdivisions (MeApd and MeApv) (Fig. 1a–c). In comparison, MeA^Foxp2^ cells are relatively fewer, constituting only 10% of MeAp cells and largely absent from caudal MeA (Fig. 1a–c). Between MeApd and MeApv, both MeA^Dbx1^ and MeA^Foxp2^ cells show a dorsal bias, with approximately twice as many cells in MeApd than MeApv (Fig. 1d). Along the medial-lateral axis of the MeApd, the MeA^Foxp2^ cells are generally located more laterally than MeA^Dbx1^ cells as reflected by their longer distances to the optic tract at multiple bregma levels (Extended Data Fig. 1a–f). Along the dorsal-ventral axis of the MeApd, the MeA^Foxp2^ cells are located more dorsally than the MeA^Dbx1^ cells (Extended Data Fig. 1g–l). Notably, consistent with our previous study, MeA^Dbx1,^ and MeA^Foxp2^ are largely distinct, even when they occupy the same MeA region (Fig. 1c–e). Of all MeA^Foxp2^ and MeA^Dbx1^ cells, only 1.8% are double positive.

### MeA^Dbx1^ and MeA^Foxp2^ cells in male mice are predominantly GABAergic

Our previous study showed that both MeAp Foxp2 and *Dbx1*-derived cells are enriched with markers for inhibitory neurons—for example, calbindin and nNOS^9,23^. To determine the neurotransmitter type of MeA^Foxp2^ cells more directly, we immunostained Foxp2 in *Vgat*^*cre*^*;Ai6*^*+/−*^ and *Vglut2*^*cre*^*;Ai6*^*+/−*^ male mice. We observed that 76% of the total MeA^Foxp2^ subpopulation is GABAergic, whereas only 5% is glutamatergic (Fig. 1f,g). Within MeAp subdivisions, there is a significantly higher percentage of Foxp2^+^Vgat^+^ double-labeled cells in the MeApd (84%) than in the MeApv (66%) and a higher percentage of MeA Foxp2^+^Vglut2^+^ cells in the MeApv (12%) than in the MeApd (2%) (Fig. 1f,h,i). We next performed in situ hybridization for Vglut2^+^ and Vgat^+^ mRNA in *Dbx1*^*cre*^*;Ai6*^*+/−*^ male mice and found that the MeA^Dbx1^ subpopulation is also primarily GABAergic. Eighty-two percent of *Dbx1*-derived cells are Vgat^+^, and only 12% are Vglut2^+^ (Fig. 1j,k). Within MeA subdivisions, 87% and 7% of MeApd *Dbx1*-derived cells are Vgat^+^ and Vglut2^+^, respectively, whereas 66% and 27% of MeApv *Dbx1*-derived cells express Vgat and Vglut2, respectively (Fig. 1j,l,m). We note that Vgat^+^ and Vglut2^+^ MeA^Dbx1^ (or MeA^Foxp2^) cells do not add up to 100%. This could be because (1) some MeA^Dbx1^ or MeA^Foxp2^ cells are non-neuronal—previous work suggested that approximately 80% of the total MeA^Foxp2^ cells express NeuN, a neuronal marker^9^; and (2) some Vgat^+^ or Vglut2^+^ cells might be missed due to their low mRNA or protein levels. Overall, these data suggest that both MeA^Dbx1^ and MeA^Foxp2^ are primarily GABAergic.

### Distinct MeA^Foxp2^ and MeA^Dbx1^ cell responses to social sensory cues

**Fig. 2:**
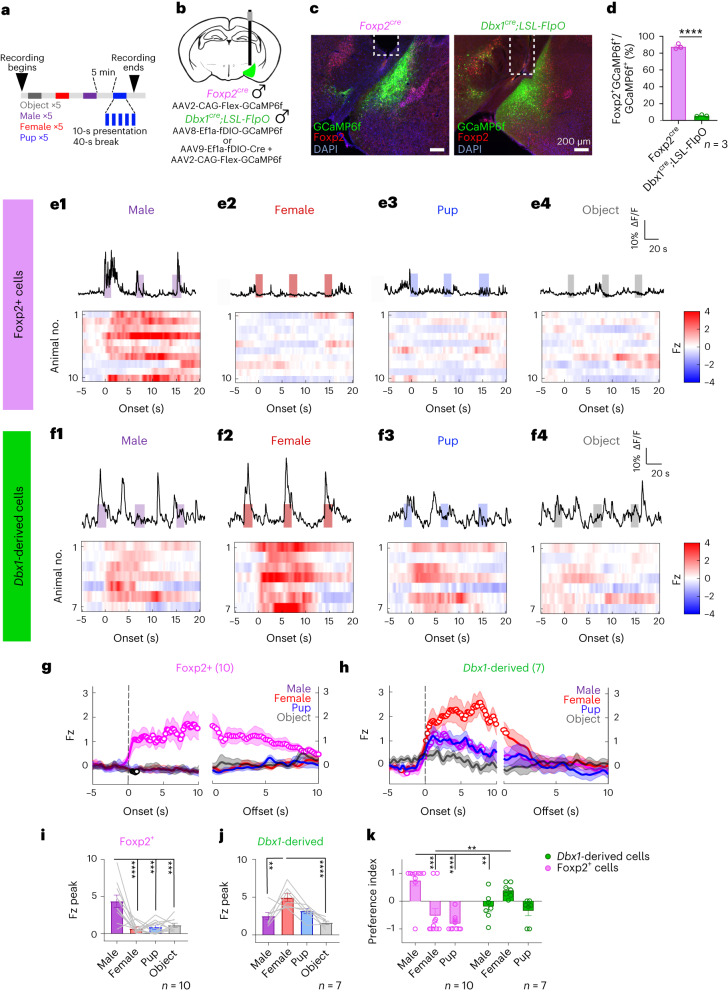
Distinct responses to social cues of MeA^Foxp2^ and MeA^Dbx1^ cells in head-fixed naive mice. **a**, Schematics showing the timeline of stimulus presentation. **b**, Schematics of viral injection strategy for targeting MeA^Foxp2^ and MeA^Dbx1^ cells. **c**, Representative histology images of viral injection, denoting GCaMP6f expression (green), Foxp2 antibody (red) and DAPI (blue) staining in *Foxp2*^*cre*^ and *Dbx1*^*cre*^*;LSL-FlpO* mice. White dotted lines mark the optic fiber tracks. **d**, Percentage of cells co-expressing Foxp2 and GCaMP6f over the total number of GCaMP6f cells in the MeA of *Foxp2*^*cre*^ and *Dbx1*^*cre*^*;LSL-FlpO* mice. **e1**–**e4**, Top, representative Ca^2+^ traces of MeA^Foxp2^ cells during the presentation of an adult male (**e1**), an adult female (**e2**), a pup (**e3**) and an object (**e4**). Colored shades represent the duration of the stimulus presentation. Bottom, corresponding heat maps of the *z*-scored Ca^2+^ responses (Fz) per animal before and after the onset of each stimulus in MeA^Foxp2^ cells. **f1**–**f4**, Responses of MeA^Dbx1^ cells to various stimuli in head-fixed naive male mice. **g**,**h**, Average peri-stimulus histograms (PSTHs) of Ca^2+^ signals from MeA^Foxp2^ (**g**) and MeA^Dbx1^ (**h**) cells aligned to the onset (left) and offset (right) of various stimulus presentations. Open circles indicate significantly increased responses (*q* < 0.05) from the baseline (Fz = 0). Colored lines and shades represent mean responses ± s.e.m. across animals. Dashed lines mark time 0. **i**,**j**, Peak Fz signal of MeA^Foxp2^ (**i**) and MeA^Dbx1^ (**j**) cells during the presentation of social and non-social stimuli. **k**, PI of MeA^Foxp2^ and MeA^Dbx1^ cells to different social stimuli. For example, PI_male_ is calculated as (Fz_male_ − 0.5 × (Fz_female_ + Fz_pup_)) / (|Fz_male_| + 0.5 × |Fz_female_ + Fz_pup_|). **d**, Unpaired *t*-test. **g**–**h**, One-sample *t*-test for each stimulus with a null hypothesis Fz = 0, corrected for repeated testing with FDR of 0.05. **i**,**j**, One-way repeated-measures ANOVA followed by Tukey’s multiple comparisons tests; *P* < 0.0001 (**i**) and *P* = 0.0004 (**j**) (interaction term). **k**, Two-way repeated-measures ANOVA followed by Sidak’s multiple comparison tests; *P* = 0.0006 (interaction term). All statistical tests are two-tailed. *n*, number of animals. Data are mean ± s.e.m; *P < 0.05, **P < 0.01, ***P < 0.001, ****P < 0.0001, otherwise *P* > 0.05. See Source Data Fig. 2 for more detailed statistics. Source data

To address whether MeA^Foxp2^ and MeA^Dbx1^ are hardwired to respond to different social cues, we recorded the Ca^2+^ activity of each population in naive adult male mice using fiber photometry while presenting various social stimuli in a pseudo-random order (Fig. 2a). Naive mice are animals without any social interaction with other conspecifics, except with their dams and littermates. To ensure that any response difference is not due to behavior differences toward different social stimuli, we head-fixed the recording animals and presented anesthetized social stimuli along a linear track so that the onset, offset and duration of the stimulus presentation were precisely controlled (Fig. 2a). To record MeA^Foxp2^ cells, we injected a GCaMP6f virus into the MeA of *Foxp2*^*cre*^ male mice^26^ (Foxp2^GCaMP^). To record MeA^Dbx1^ cells, we generated *Dbx1*^*cre*^*;LSL-FlpO* mice. In these animals, the transient Cre expression during embryogenesis, when Dbx1 is expressed, drives permanent Flp expression, allowing targeting of *Dbx1*-derived cells in adult mice^27^. We then injected a GCaMP6f virus into the MeA of *Dbx1*^*cre*^*;LSL-FlpO* male mice (Dbx1^GCaMP^) (Fig. 2b). Histological analysis revealed that 88% of GCaMP6f cells express Foxp2 in Foxp2^GCaMP^ mice, whereas only 5% of GCaMP6f cells were co-labeled with Foxp2 in Dbx1^GCaMP^ mice, confirming the specificity of the recorded populations (Fig. 2c,d).

MeA^Foxp2^ cells in naive male mice showed robust GCaMP6 increases only during the presentation of adult males but not adult females, pups or objects (Fig. 2e,g,i,k). In contrast, MeA^Dbx1^ cells showed the highest activity increase during the adult female presentation (Fig. 2f,h,j,k). MeA^Foxp2^ and MeA^Dbx1^ cells also differed in their response dynamics. MeA^Foxp2^ cells returned to the baseline activity slowly (>10 s) after the removal of the male stimulus, whereas the MeA^Dbx1^ cell activity returned to the baseline quickly (<3 s) (Fig. 2g,h). Overall, MeA^Foxp2^ cells showed male-specific and slow decaying responses, whereas MeA^Dbx1^ cells showed broad responses to social cues (Fig. 2g–k). These results strongly support distinct response patterns of MeA^Foxp2^ and MeA^Dbx1^ cells to social stimuli independent of fighting or mating experience.

### Distinct MeA^Dbx1^ and MeA^Foxp2^ cell responses during social behaviors

**Fig. 3:**
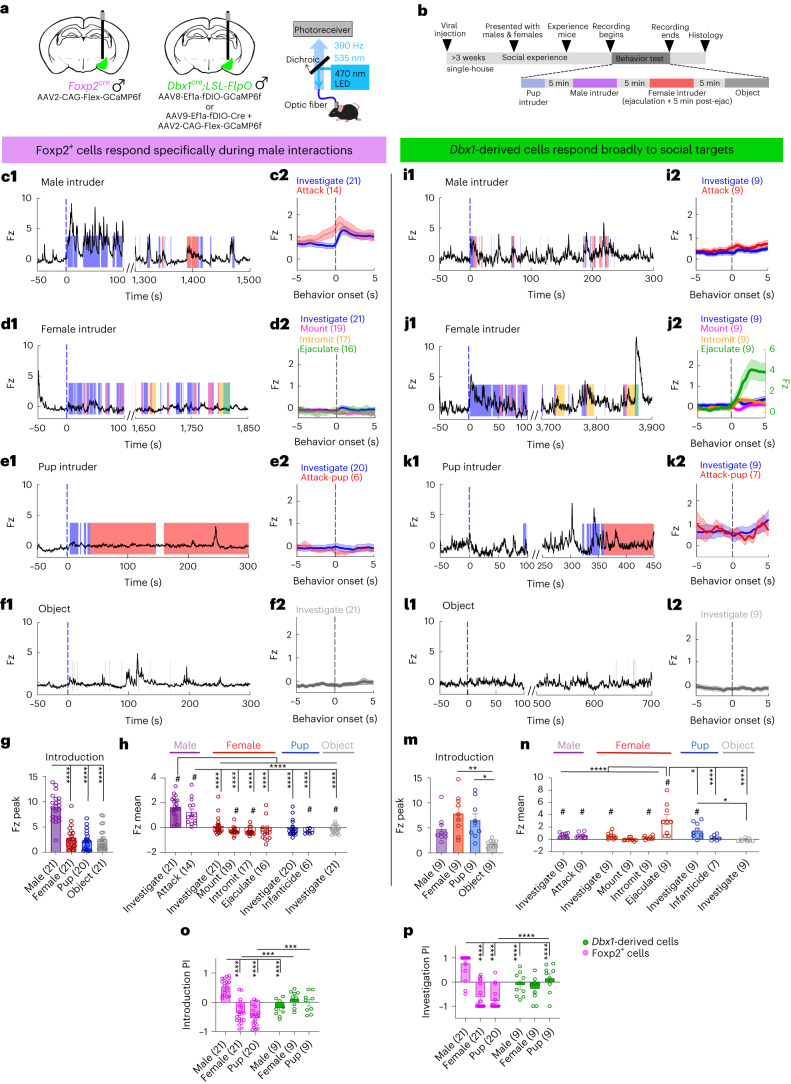
Differential response patterns of MeA^Foxp2^ and MeA^Dbx1^ cells during fighting and mating in socially experienced male mice. **a**, Schematics of viral strategies and the fiber photometry setup. **b**, Experimental timeline for Ca^2+^ recordings in freely moving experienced male mice. **c**,**f**,**i**–**l**, Representative Ca^2+^ traces and PETHs of MeA^Foxp2^ (**c**–**f**) and MeA^Dbx1^ (**i**–**l**) cells during interactions with an adult male, an adult female, a pup and an object. Dashed black lines in PETHs represent the behavior onset at time 0; blue lines in Ca^2+^ traces indicate time 0 when the intruder is introduced. **g**,**m**, Introduction responses of MeA^Foxp2^ (**g**) and MeA^Dbx1^ (**m**) cells, calculated as the peak Ca^2+^ signal within the first 100 s after stimulus introduction. **h**,**n**, Average Ca^2+^ responses of MeA^Foxp2^ (**h**) and MeA^Dbx1^ (**n**) cells during behaviors toward various conspecific intruders and a novel object. **o**, PIs of MeA^Foxp2^ and MeA^Dbx1^ cells showing the relative introduction response magnitudes across different social stimuli. **p**, PIs of MeA^Foxp2^ and MeA^Dbx1^ cells showing the relative investigation response magnitudes across different social stimuli. **g**,**h**,**n**–**p**, Mixed-effects analysis followed by Sidak’s multiple comparisons tests; *P* < 0.0001 (interaction term). **m**, Friedman test followed by FDR correction; *P* = 0.0023. **h**,**n**, One-sample *t*-test with null hypothesis Fz = 0, corrected for repeated testing with FDR of 0.05. Parentheses indicate number of animals. All statistical tests are two-tailed. Data are mean ± s.e.m; *P < 0.05, **P < 0.01, ***P < 0.001, ****P < 0.0001, #*q* < 0.05, otherwise *P* > 0.05 and *q* > 0.05. See Source Data Fig. 3 for more detailed statistics. Source data

**Extended Data Fig. 2:**
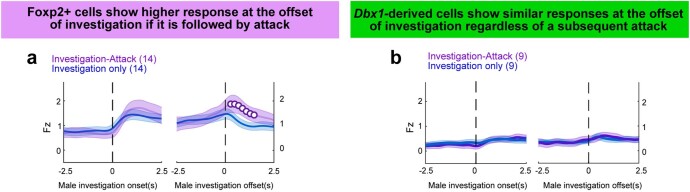
Additional characterization of MeA^Foxp2^ and MeA^Dbx1^ cell responses in experienced male mice, related to Fig. 3. **(a)** Average PETHs of MeA^Foxp2^ Ca^2+^ signal aligned to the onset (left) and offset (right) of investigation only (blue) and investigation-followed-by-attack (purple) trials. Open circles denote the period with significantly different responses (p < 0.05). **(b)** Average PETHs of MeA^Dbx1^ cell responses aligned to the onset (left) and offset (right) of investigation only (blue) and investigation-followed-by-attack (purple) trials. The two traces do not differ significantly at any time point. (**a** and **b**) Two-tailed one-sample t-test at each time point corrected for repeated testing with FDR 0.05. Parentheses denote the number of animals. Data are mean ± SEM. See Source Data Extended Data Fig. 2 for more detailed statistics. Source data

**Extended Data Fig. 3:**
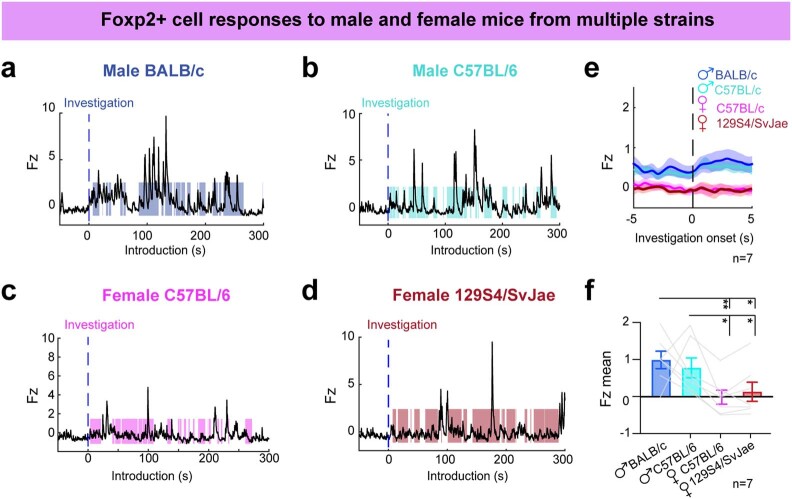
MeA^Foxp2^ cell responses to male and female mice from multiple strains, related to Fig. 3. **(a-d)** Representative Ca^2+^ traces of MeA^Foxp2^ cells during the presentation of a BALB/c male **(a)**, a C57BL/6 male **(b)**, a C57BL/6 female **(c)**, and a 129S4/SvJae female **(d)** mouse. **(e)** Average PETHs of MeA^Foxp2^ cell responses aligned to investigation onset of mice from different strains. The dashed black line represents the behavior onset at time zero. **(f)** Average Fz scores of MeA^Foxp2^ cells during investigation of male and female mice from different strains; p = 0.0052. Friedman test followed by multiple comparison tests with correction for FDR of 0.05. n = number of animals. Data are mean ± SEM; *p < 0.05, **p < 0.01. Otherwise, p > 0.05. See Source Data Extended Data Fig. 3 for more detailed statistics. Source data

Next, we examined responses of male MeA^Foxp2^ and MeA^Dbx1^ cells during social behaviors in freely moving male mice to address whether the cells increase activity only to sensory cues—for example, during investigation—or also during the action phase of the behavior—for example, attack and mount (Fig. 3a). Before recording, all test animals went through up to 12 interactions with an adult male and a female (once per day) to ensure behavior stability. During recording, a non-aggressive adult BALB/c male intruder, a sexually receptive female, a pup and a novel object were introduced into the home cage of the recorded mice, one at a time, with 5 min in between (Fig. 3b). MeA^Foxp2^ cells significantly increased activity upon introducing a male, more than the responses during the introduction of any other social and non-social stimuli, and the activity remained elevated when the male was present (Fig. 3c–h). During each episode of male investigation and attack, MeA^Foxp2^ cells also showed a significant activity increase (Fig. 3h). To address whether the activity increase during attack simply reflects the elevated activity during investigation, we separated investigation trials based on whether they were followed by attack or not. We found that MeA^Fxop2^ cell activity at the offset of investigation is higher in investigation-followed-by-attack trials than investigation-not-followed-by-attack trials, suggesting that the elevated activity during attack is not simply due to sensory inputs during the preceding investigation (Extended Data Fig. 2a). In contrast to the strong response during male interaction, MeA^Foxp2^ cells showed either no change or slightly suppressed activity during female investigation and all phases of sexual behaviors (Fig. 3d,h). Similarly, no activity change was observed during pup interaction, supporting a highly adult male-specific response of MeA^Foxp2^ cells (Fig. 3e,h). Notably, the adult male-specific response of MeA^Foxp2^ cells is not limited to one particular strain. We found that BALB/c and C57BL/6 males evoked similarly strong responses, significantly higher than those evoked by C57BL/6 or 129S4/SvJae females (Extended Data Fig. 3a–f).

In contrast to the response pattern of MeA^Foxp2^ cells, MeA^Dbx1^ cells in experienced male mice showed activity increases to all social stimuli (Fig. 3i–k). Upon initial intruder introduction, MeA^Dbx1^ cells increased activity regardless of the identity of the intruder, and the responses to adult females and pups were significantly higher than those to objects (Fig. 3m). During investigation, MeA^Dbx1^ cell activity also increased to a similar extent to all social targets (Fig. 3n). Although MeA^Dbx1^ cells showed significant activity increase during inter-male attack, we did not find the responses during investigation-followed-by-attack trials and those during investigation-only trials to differ, suggesting that MeA^Dbx1^ cell activity during attack could be largely due to activity increases during investigation preceding attack (Fig. 3i,n and Extended Data Fig. 2b). During copulation, the activity of MeA^Dbx1^ cells did not increase during mounting but slightly increased during intromission (Fig. 3j,n). During ejaculation, MeA^Dbx1^ cells increased activity robustly, significantly higher than the responses during any other behaviors (Fig. 3j,n). No activity increase of MeA^Dbx1^ cells was observed when males attacked pups (Fig. 3k,n). MeA^Dbx1^ cells did not respond during object investigation, whereas MeA^Foxp2^ cells showed a slight suppression in activity during object investigation (Fig. 3f,h,l,n), supporting the social-specific response patterns of the cells.

**Extended Data Fig. 4:**
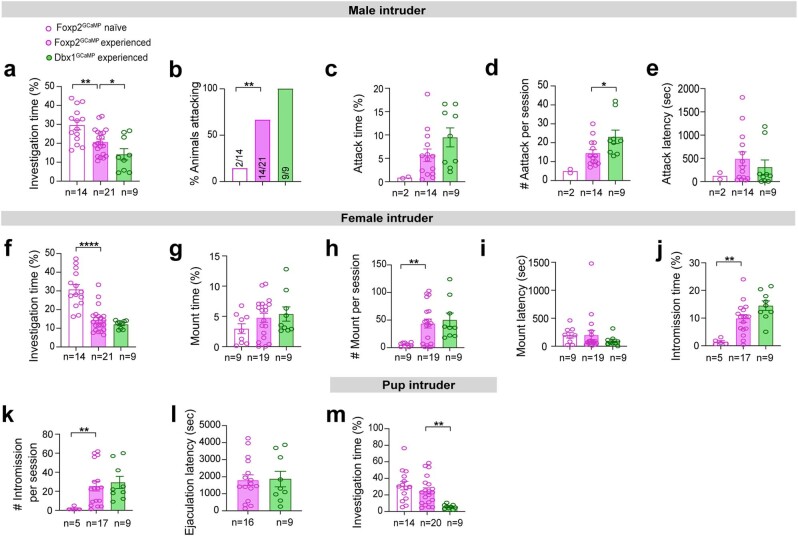
Quantification of behaviors towards different intruders in Foxp2^GCaMP^ and Dbx1^GCaMP^ male mice, related to Fig. 3. **(a)** Percent of time naïve Foxp2^GCaMP^, socially experienced Foxp2^GCaMP^ and socially experienced Dbx1^GCaMP^ male mice spent investigating a male intruder. **(b)** The fraction of naïve Foxp2^GCaMP^, socially experienced Foxp2^GCaMP^ and socially experienced Dbx1^GCaMP^ male mice that attacked a male intruder. **(c-e)** Percent of time naïve Foxp2^GCaMP^, socially experienced Foxp2^GCaMP^ and socially experienced Dbx1^GCaMP^ male mice spent attacking a male intruder **(c)**, as well as the latency to first attack **(d)** and total number of attack events **(e). (f-l)** Percent of time Foxp2^GCaMP^ and Dbx1^GCaMP^ male mice spent investigating **(f)**, mounting **(g)**, and intromitting **(j)** a receptive female intruder, as well as the total number of mounting events per recording session **(h)**, latency to first mount **(i)**, total number of intromission events **(k)** and latency to ejaculation **(l)**. **(m)** Percent of time Foxp2^GCaMP^ and Dbx1^GCaMP^ male mice spent investigating a pup intruder. **(a, c-m)** Two-tailed unpaired t-tests (if pass normality test) or two-tailed Mann Whitney tests (if not pass normality test) between naïve Foxp2^GCaMP^ and socially experienced Foxp2^GCaMP^ animals, and between socially experienced Foxp2^GCaMP^ and Dbx1^GCaMP^ animals; **(d)** p = 0.0148, **(f)** p < 0.0001, **(h)** p = 0.0039 between naïve Foxp2^GCaMP^ and socially experienced Foxp2^GCaMP^ animals, **(j)** p = 0.0045 between naïve Foxp2^GCaMP^ and socially experienced Foxp2^GCaMP^ animals, **(k)** p = 0.0016 between naïve Foxp2^GCaMP^ and socially experienced Foxp2^GCaMP^ animals, **(m)** p = 0.0025 between socially experienced Foxp2^GCaMP^ and socially experienced Dbx1^GCaMP^ male mice. **(b)** Two-tailed Chi-square tests; p = 0.0023 between naïve Foxp2^GCaMP^ and socially experienced Foxp2^GCaMP^ animals. n = number of animals. Data are mean ± S.E.M.; *p < 0.05, **p < 0.01, ***p < 0.001. Otherwise, p > 0.05. See Source Data Extended Data Fig. 4 for more detailed statistics. Source data

The sexual and aggressive behaviors of MeA^Foxp2^ and MeA^Dbx1^ male mice are largely similar, except that MeA^Dbx1^ attacked more frequently (Extended Data Fig. 4). Thus, changes in neuronal activity observed during recordings are unlikely due to behavioral differences. Overall, male MeA^Foxp2^ cells show highly specific responses during both the investigatory and action phases of behaviors toward a conspecific adult male, whereas MeA^Dbx1^ cells respond to diverse social cues and during ejaculation.

### Refinement of MeA^Foxp2^ cell responses with adult social experience

**Fig. 4:**
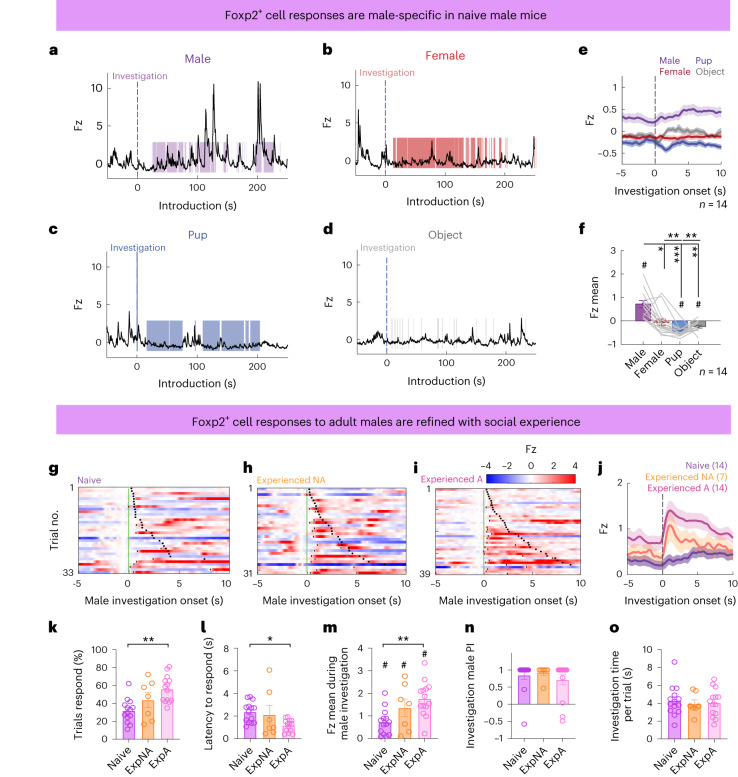
Comparison of MeA^Foxp2^ cell responses in naive versus non-aggressive and aggressive experienced male mice. **a**–**d**, Representative Ca^2+^ traces of MeA^Foxp2^ cells during the presentation of an adult male (**a**), an adult female (**b**), a pup (**c**) and an object (**d**) in naive male mice. **e**, Average PETHs of MeA^Foxp2^ cell responses aligned to investigation onset in naive male mice. The dashed black line represents the behavior onset at time 0. **f**, Average Fz score of MeA^Foxp2^ cells during investigation of different stimuli in naive male mice. **g**–**i**, Representative heat maps showing trial-by-trial Ca^2+^ signal in normalized Fz (by subtracting the signal at time 0) of MeA^Foxp2^ cells while investigating a male intruder in a naive (**g**), experienced non-aggressive (experienced NA) (**h**) and experienced aggressive (experienced A) (**i**) male mouse. Black short lines denote the timepoints when Fz ≥ 1. Black dots denote the investigation offsets. **j**, Average PETHs of MeA^Foxp2^ cell responses aligned to investigation onset in naive (purple), experienced NA (orange) and experienced A (pink) male mice. **k**, Percent of male investigation trials in which MeA^Foxp2^ cells reach Fz ≥ 1. **l**, Latency of MeA^Foxp2^ cells to respond (Fz > 1) in responsive trials. **m**, Average Fz score of MeA^Foxp2^ cells during male investigation. **n**, Male PIs of MeA^Foxp2^ cell responses during investigation across experience. **o**, Average male investigation duration per trial. **f**, Friedman test followed by multiple comparison tests with FDR correction; *P* = 0.0006. One-sample *t*-test for each stimulus with null hypothesis Fz = 0, corrected for repeated testing with FDR of 0.05. **k**–**m**,**o**, One-way ANOVA followed by Tukey’s multiple comparison tests; *P* = 0.0013 (interaction term) (**k**), *P* = 0.0278 (interaction term) (**l**), *P* = 0.0037 (interaction term) (**m**). **m**, One-sample *t*-test with null hypothesis Fz = 0, corrected for repeated testing with FDR of 0.05. **n**, Kruskal–Wallis test followed by the multiple comparison tests with FDR correction. Parentheses and *n* indicate the number of animals per group. **k**–**o**, Naive group *n* = 14; Experienced NA group *n* = 7; Experienced A group *n* = 14. All statistical tests are two-tailed. Data are mean ± s.e.m. #*q* < 0.05, *P < 0.05, **P < 0.01, ***P < 0.001, otherwise *P* > 0.05 and *q* > 0.05. See Source Data Fig. 4 for more detailed statistics. Source data

In some Foxp2^GCaMP^ animals, we performed Ca^2+^ recordings during freely moving social interactions before repeated social experiences. Two of 14 naive male Foxp2^GCaMP^ mice briefly attacked a male intruder in the 10-min testing period, and others only investigated the intruders (Extended Data Fig. 4a,b). Similar to our recordings in head-fixed naive animals, MeA^Foxp2^ cells responded specifically during male investigation (Fig. 4a–f). We then directly compared MeA^Foxp2^ cell responses between naive and experienced animals. The experienced animals were divided into two groups: mice that attacked a male intruder in the recording session (experienced aggressors) and those that did not (experienced non-aggressors).

The activity of MeA^Foxp2^ cells in experienced aggressors increased faster and with higher consistency during male investigation than in naive animals (Fig. 4g–j). MeA^Foxp2^ cells responded (Z_increase_ > 1 during investigation) in approximately 32% of trials in naive animals. In comparison, this number increased to 55% in experienced aggressors (Fig. 4k). Among the responsive trials, the average latency to respond in experienced aggressors is approximately half of that in naive animals (Fig. 4l). The mean activity increase during male investigation is significantly higher in experienced aggressors than in naive animals (Fig. 4m). The MeA^Foxp2^ cell responses in experienced non-aggressors generally fell in between those in naive and experienced aggressors. Nevertheless, the male preference index (PI) did not differ among these three groups (Fig. 4n). Furthermore, the average duration per investigation episode was similar across the groups (Fig. 4o). These results suggest that, although adult aggressive experience is not required for the male-specific responses of MeA^Foxp2^ cells, it refines the response by improving its trial-to-trial consistency and temporal precision.

### The male-specific response of MeA^Foxp2^ cells exists before puberty

**Fig. 5:**
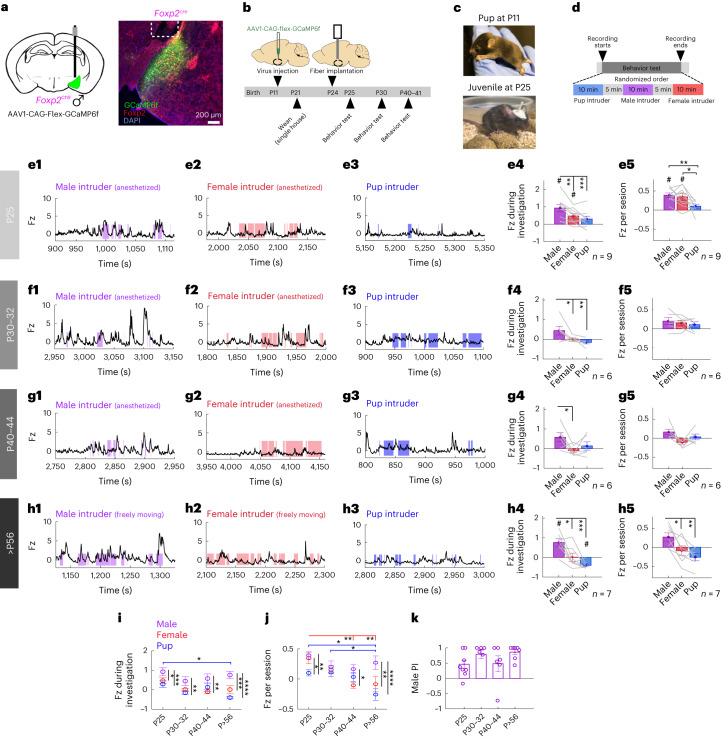
MeA^Foxp2^ cell responses before, during and after puberty in developing male mice. **a**, Schematics of virus injection and a representative histology image indicating GCaMP6f expression (green), Foxp2 antibody (red) and DAPI (blue) staining in *Foxp2*^*cre*^ male mice. White dotted lines mark the fiber end. **b**, Timeline of virus injection, fiber placement and recordings. **c**, Pup at P11 before viral surgery and juvenile at P25 before experimental recording. **d**, Timeline of behavioral test during the recording day. Stimuli were presented in a pseudo-random order. **e**–**h,** Representative Fz-scored Ca^2+^ traces of MeA^Foxp2^ cells during interactions with an anesthetized (**e1**–**g1**) or freely moving (**h1**) male, an anesthetized (**e2**–**g2**) or freely moving (**h2**) female or a pup (**e3**–**h3**) in a male mouse at different ages. Average Fz score during social investigation (**e4**–**h4**) of animals at different ages. Average Fz score of the entire intruder session (**e5**–**h5**) of animals at different ages. **i**, Average Fz score of MeA^Foxp2^ cell responses during male (purple), female (red) and pup (blue) investigation in male mice of different ages. **j**, Average Fz score of MeA^Foxp2^ cells per intruder session at different ages. **k**, Male investigation PIs at different ages. **e4**–**h4**,**e5**–**h5**, One-way repeated-measures ANOVA followed by Tukey’s multiple comparison tests; interaction terms: *P* = 0.0003 (**e4**), *P* = 0.0071 (**e5**), *P* = 0.0084 (**f4**), *P* = 0.0295 (**g4**), *P* = 0.0009 (**h4**), *P* = 0.0026 (**h5**). One-sample *t*-test for each behavior with null hypothesis Fz = 0, corrected for repeated testing with FDR of 0.05. **i**,**j**, Two-way repeated-measures ANOVA followed by Sidak’s multiple comparison tests; *P* = 0.1007 (interaction term) (**i**) and *P* = 0.0137 (interaction term) (**j**). The color of the line specifies the social stimulus that evokes significantly different responses over development. **k**, Kruskal–Wallis test followed by multiple comparison tests with FDR correction. *n* = 9 (P25), 6 (P30–P32), 6 (P40–P44) and 7 (>P56) mice. *n*, number of animals. All statistical tests are two-tailed. Data are mean ± s.e.m. *P < 0.05, **P < 0.01, ***P < 0.001, ****P < 0.0001, #*q* < 0.05, otherwise *P* > 0.05 and *q* > 0.05. See Source Data Fig. 5 for more detailed statistics. Source data

**Extended Data Fig. 5:**
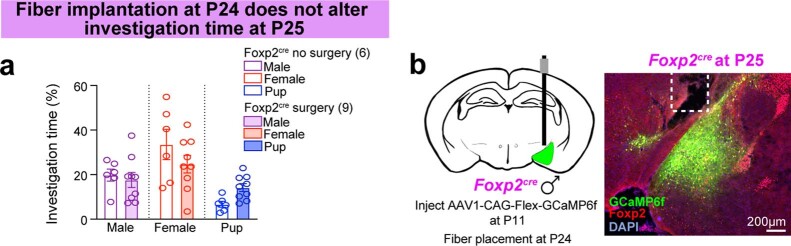
GCaMP viral expression and investigation time in Foxp2^cre^ mice at P25, related to Fig. 5. **(a)** Percent of time P25 *Foxp2*^*cre*^ mice spent investigating an anaesthetized male, anaesthetized female or pup intruder after fiber implantation surgery at P24 or without surgery. Two-way ANOVA followed by Sidak’s multiple comparison tests. All p values > 0.05. Parentheses denote the number of animals. Data are mean ± SEM. **(b)** Schematic of virus injection and a representative histology image indicating GCaMP6f expression (green), Foxp2 antibody (red), and DAPI (blue) staining in Foxp2^cre^ male mice at P25. White dotted lines mark the fiber end. See Source Data Extended Data Fig. 5 for more detailed statistics. Source data

To further address whether the male-specific MeA^Foxp2^ cell responses are developmentally hardwired or established through adult experience, we recorded the responses of MeA^Foxp2^ cells to social stimuli during early life. Puberty (P30–P38) is a critical development period when aggression emerges^28–30^. Thus, we focused on MeA^Foxp2^ cell responses before puberty (P25), at the onset of puberty (P30–P32) and after puberty (P40–P44). To achieve this goal, we injected Cre-dependent GCaMP6f virus into the MeA of P11 *Foxp2*^*cre*^ mice and placed a 400-µm fiber just dorsal to the MeA at P24 and allowed for 24-h recovery before recording at P25 (Fig. 5a–c). Behaviorally, recorded juvenile *Foxp2*^*cre*^ mice (P25) spent a similar amount of time investigating the intruders as age-matched *Foxp2*^*cre*^ mice that had not undergone surgery, suggesting sufficient recovery (Extended Data Fig. 5a). Histological analysis confirmed high levels of GCaMP6 expression at P25 (Extended Data Fig. 5b).

We recorded the Ca^2+^ activity of MeA^Foxp2^ cells when the animals were exposed to an anesthetized adult male and adult female mouse or a pup at P25, P30–32 and P40–P44 (Fig. 5d). To minimize the impact of social experience, all animals were singly housed after weaning at P21. We found that MeA^Foxp2^ cells in P25 juvenile male mice already showed higher activity during close interaction with an adult male than an adult female or pups (Fig. 5e). However, when we consider the average activity of the entire recording session, the presence of either an adult male or female, but not a pup, caused an elevation in GCaMP6 activity (Fig. 5e5). At P30–P32, a similar male-biased response was observed during close interaction, whereas the overall GCaMP6 activity during the recording session was not significantly elevated regardless of the intruder (Fig. 5f5). At P40–P44, the difference between male and female responses during investigation increased (Fig. 5g), and this trend continued at >P56 (Fig. 5h). At >P56, the presence of a male caused a significant elevation in the GCaMP6 activity, whereas female and pup presence caused either no change or slightly suppressed activity (Fig. 5h,i). As a result of gradually decreased activity to non-male social cues, the MeA^Foxp2^ cells become increasingly tuned to adult males over development (Fig. 5j), although male-biased responses are seen at all ages (Fig. 5k). Altogether, these results suggest that MeA^Foxp2^ cells are predisposed to preferentially responding to male-related sensory information even before puberty, and the discriminability between adult male and non-male cues is further refined after puberty by reducing responses to non-adult male cues.

### Differential inputs to MeA^Foxp2^ and MeA^Dbx1^ cells

**Fig. 6:**
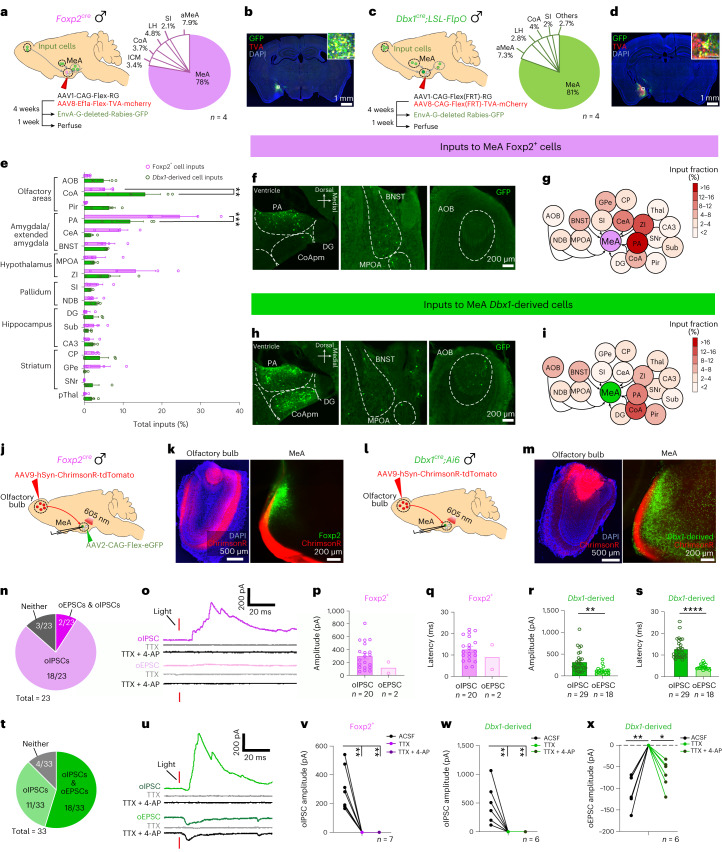
Differences in the anatomical and functional inputs of MeA^Foxp2^ and MeA^Dbx1^ cells for sensory processing. **a**,**c**, The timeline of monosynaptic retrograde rabies tracing of MeA^Foxp2^ (**a**) and MeA^Dbx1^ (**c**) cells and distribution of starter cells (mCherry^+^GFP^+^). **b**,**d**, Representative images showing the location of starter MeA^Foxp2^ cells in a *Foxp2*^*cre*^ mouse (**b**) or MeA^Dbx1^ cells in a *Dbx1*^*cre*^*;LSL-FlpO* mouse (**d**). TVA-mCherry (red), Rabies-GFP (green) and DAPI (blue) staining. Scale bars, 100 µm (top right). **e**, Distribution of retrogradely labeled cells. **f**,**h**, Representative histological images with cells retrogradely labeled from MeA^Foxp2^ (**f**) and MeA^Dbx1^ (**h**) cells. **g**,**i**, Overview of inputs into MeA^Foxp2^ (**g**) and MeA^Dbx1^ (**i**) cells. **j**,**l**, Recording strategy examining synaptic inputs from the AOB to MeA^Foxp2^ (**j**) and MeA^Dbx1^ (**l**) cells. **k**,**m**, Representative images showing ChrimsonR (red) expression in the olfactory bulb (OB) and ChrimsonR fibers in the MeA. Green: GFP expressed in Foxp2 (**k**) and Dbx1 (**m**) cells. Blue: DAPI staining. **n**,**t**, The distribution of synaptic responses of MeA^Foxp2^ (**n**) and MeA^Dbx1^ (**t**) cells to OB terminal activation. **o**,**u**, Representative traces showing optogenetically (1 ms, 605 nm) evoked IPSCs (oIPCSs) and EPSCs (oEPSCs) before and after bath application of TTX and TTX + 4-AP. **p**–**s**, Amplitude (**p**,**r**) and latency (**q**,**s**) of oIPSCs and oEPSCs in MeA^Foxp2^ and MeA^Dbx1^ cells. **v**–**w,** oIPSCs in MeA^Foxp2^ (**v**) and MeA^Dbx1^ (**w**) cells were abolished by TTX and failed to recover after applying TTX + 4-AP. **x**, oEPSCs in MeA^Dbx1^ cells were abolished by TTX but recovered after TTX + 4-AP application. **a**,**c**, *n*, number of animals for **a**–**i**. **e**, Two-way ANOVA followed by Sidak’s multiple comparison tests; *n* = 4 mice per group; *P* < 0.0001. **r**,**s**, Mann–Whitney test; *P* = 0.0002 (**r**) and *P* < 0.0001 (**s**). **v**–**x,** Friedman test followed by multiple comparison tests with FDR correction; *P* = 0.0014, *n* = 7 cells from three male mice (**v**); *P* = 0.0041 and *P* = 0.0017, respectively (**w**,**x**), *n* = 6 cells from three male mice. **n**–**s,** *n* = 23 cells from three male mice for MeA^Foxp2^ group and *n* = 33 cells from three male mice for MeA^Dbx1^group, where *n* is the number used for statistical analysis. All statistical tests are two-tailed. Data are mean ± s.e.m. *P < 0.05, **P < 0.01, ***P < 0.001, ****P < 0.0001, otherwise *P* > 0.05. See Extended Data Table 1 for brain region abbreviations. See Source Data Fig. 6 for more detailed statistics. Source data

Given the differential responses of MeA^Foxp2^ and MeA^Dbx1^ cells to social cues, we next asked whether these two populations receive inputs from different brain regions by performing monosynaptic rabies virus tracing. We injected Cre-dependent or Flp-dependent adeno-associated viruses (AAVs) expressing TVA-mCherry and rabies G protein into the MeA of *Foxp2*^*cre*^ or *Dbx1*^*cre*^*;LSL-FlpO* male mice and, 4 weeks later, EnvA-ΔG rabies virus expressing GFP (Fig. 6a–d). We found that the major inputs to MeA^Foxp2^ are from other amygdala nuclei, including the posterior amygdala (PA), central amygdala (CeA) and BNST (Fig. 6e–g). In contrast, MeA^Dbx1^ cells receive inputs mainly from primary olfactory relays, including AOB, cortical amygdala (CoA) and the piriform cortex (Pir) (Fig. 6e,h,i). Hypothalamus, mainly the medial preoptic area (MPOA) and zona incerta (ZI), provided moderate inputs to both MeA^Foxp2^ and MeA^Dbx1^ cells (Fig. 6e–i). Sparsely retrogradely labeled cells from both MeA^Foxp2^ and MeA^Dbx1^ cells were also observed in the hippocampus, striatum and pallidum (Fig. 6e–i).

The lack of retrogradely labeled cells in the AOB from MeA^Foxp2^ starter cells was particularly surprising given that the MeA is the primary target of the AOB (Fig. 6e–g)^4,31,32^. To further dissect the inputs from the AOB to MeA^Foxp2^ cells, we performed optogenetic-assisted circuit mapping. We expressed ChrimsonR-tdTomato in the olfactory bulb, virally labeled MeA^Foxp2^ cells with GFP (Fig. 6j,k) and visualized MeA^Dbx1^ cells using *Dbx1*^*cre*^*;Ai6* mice (Fig. 6l,m). Four weeks after injection, we prepared brain slices containing the MeA and recorded the responses of GFP^+^ MeA^Foxp2^ and MeA^Dbx1^ cells to 605-nm, 1-ms light pulses. Among 23 recorded MeA^Foxp2^ cells, we observed light-evoked excitatory postsynaptic currents (oEPSCs) in only two cells, and 18 of 23 recorded cells showed light-evoked inhibitory postsynaptic currents (oIPSCs) (Fig. 6n,o). In contrast, 18 of 33 MeA^Dbx1^ cells showed oEPSCs, and 29 of 33 showed oIPSCs (Fig. 6t,u). The oIPSCs of MeA^Dbx1^ and MeA^Foxp2^ cells were similar in magnitude, and both were of long latencies (>10 ms) (Fig. 6p–s). Bath application of TTX or TTX + 4-AP completely abolished oIPSCS in both populations, suggesting that they are polysynaptic connections (Fig. 6o,u–w). oEPSCs of MeA^Dbx1^ cells were of short latency (~4 ms) (Fig. 6s), and their amplitude did not change after TTX + 4-AP application, supporting that AOB cells provide monosynaptic excitatory inputs to MeA^Dbx1^ cells (Fig. 6u,x).

These results confirmed that AOB targets MeA^Foxp2^ and MeA^Dbx1^ cells differently. The fact that MeA^Foxp2^ cells receive minimum direct inputs from the AOB and other primary olfactory relays suggests that sensory information reaching MeA^Foxp2^ cells is likely more processed, which may explain the higher response selectivity of MeA^Foxp2^ cells than MeA^Dbx1^ cells.

### Activating MeA^Foxp2^ cells is sufficient for aggression in naive mice

**Fig. 7:**
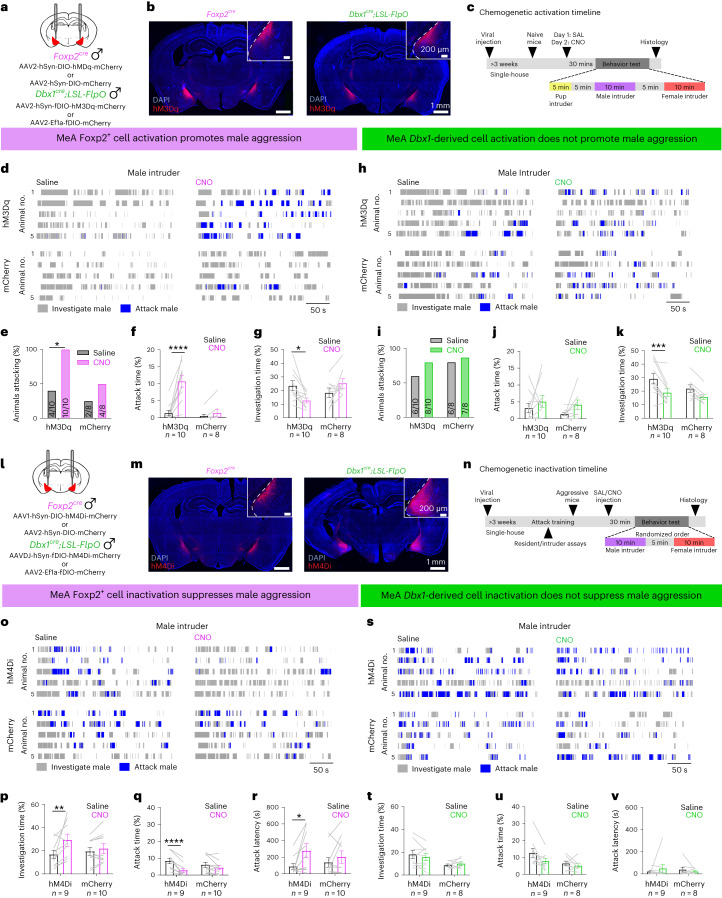
MeA^Foxp2^ cells bi-directionally modulate territorial aggression, whereas MeA^Dbx1^ cells do not. **a**, Strategies for chemogenetic activation of MeA^Foxp2^ and MeA^Dbx1^ cells. **b**, Representative histological images of hM3Dq (red) expression in the MeA of *Foxp2*^*cre*^ and *Dbx1*^*cre*^*;LSL-FlpO* mice. Top right shows an enlarged view of the MeA infection. Blue: DAPI. **c**, Experimental timeline of chemogenetic activation experiments. **d**, Representative raster plots showing behaviors toward male intruders of five Foxp2^hM3Dq^ and five Foxp2^mCherry^ male mice after i.p. injection of saline or CNO. **e**, Percentage of Foxp2^hM3Dq^ and Foxp2^mCherry^ male mice that attacked a male intruder after saline or CNO injection. **f**,**g**, Percent of time Foxp2^hM3Dq^ and Foxp2^mCherry^ mice spent attacking (**f**) and investigating (**g**) a male intruder. **h**–**k**, Follows the conventions in **d**–**g**. CNO injection into Dbx1^hM3Dq^ mice caused a reduction in social investigation but did not change aggressive behaviors toward a male intruder. **l**, Strategies for chemogenetic inactivation of MeA^Foxp2^ and MeA^Dbx1^ cells. **m**, Representative histological images showing hM4Di (red) expression in the MeA of *Foxp2*^*cre*^ and *Dbx1*^*cre*^*;LSL-FlpO* mice. Top right shows an enlarged view of the MeA infection. Blue: DAPI. **n**, Experimental timeline of chemogenetic inactivation experiments. **o**, Representative raster plots showing behaviors toward male intruders of five Foxp2^hM4Di^ and five Foxp2^mCherry^ mice after i.p. injection of saline or CNO. **p**–**r**, Percent of time Foxp2^hM4Di^ and Foxp2^mCherry^ male mice spent investigating (**p**) and attacking (**q**) a male intruder and the latency to first attack (**r**). **s**–**v**, Follows the conventions in **o**–**r**. CNO injection into Dbx1^hM4Di^ or Dbx1^mCherry^ mice did not change any male-directed behaviors in comparison to those after saline injection. **e**,**i,** McNemar’s test; *P* = 0.0412 (**e**). **f**,**g**,**j**,**k**,**p**–**r**,**t**–**v**, Two-way repeated-measures ANOVA followed by Sidak’s multiple comparison tests; interaction terms: *P* = 0.0004 (**f**), *P* = 0.004 (**g**), *P* = 0.2653 (**k**), *P* = 0.0550 (**p**), *P* = 0.0091 (**q**) and *P* = 0.1836 (**r**). *n*, number of animals. All statistical tests are two-tailed. Data are mean ± s.e.m. *P < 0.05, **P < 0.01, ***P < 0.001, ****P < 0.0001, otherwise *P* > 0.05. See Source Data Fig. 7 for more detailed statistics. Source data

**Extended Data Fig. 6:**
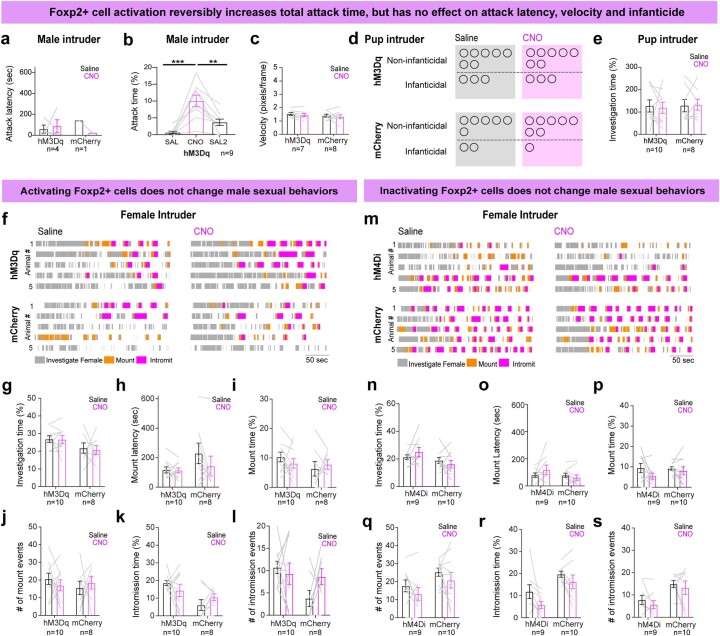
Additional behavioral assays after chemogenetic activation and inactivation of MeA^Foxp2^ cells, related to Fig. 7. **(a)** In Foxp2^hM3Dq^ and Foxp2^mCherry^ male mice, the latency to attack a male intruder after CNO injection did not differ from that after saline injection. Only animals that showed attack after both saline and CNO injections were included in this analysis. **(b)** Percent of time Foxp2^hM3Dq^ male mice spent attacking a male intruder during three consecutive days of testing after 1^st^ day i.p. saline injection (SAL), 2^nd^ day CNO injection (CNO), and 3^rd^ day saline injection (SAL2). **(c)** No changes in velocity (pixels/frame) in Foxp2^hM3Dq^ or Foxp2^mCherry^ male mice were observed in a 5 min period 30 min after CNO or saline injection when the test animal was alone in its cage. **(d)** Number of Foxp2^hM3Dq^ or Foxp2^mCherry^ male mice that attacked pups vs. those that did not after saline or CNO injection. Each circle represents one mouse. **(e)** Percentage of time Foxp2^hM3Dq^ or Foxp2^mCherry^ male mice spent investigating the pup after saline or CNO injection. **(f)** Representative raster plots showing the behaviors towards an adult female intruder of 5 Foxp2^hM3Dq^ and 5 Foxp2^mCherry^ mice after i.p. injection of saline or CNO. **(g-l)** Between CNO-injected and saline-injected days, there is no difference in any parameters related to male sexual behaviors in Foxp2^mCherry^ and Foxp2^hM3Dq^ male mice. **(m)** Representative raster plots showing the behaviors towards an adult female intruder of 5 Foxp2^hM4Di^ and 5 Foxp2^mCherry^ mice after i.p. injection of saline or CNO. **(n-s)** Between CNO-injected and saline-injected days, there is no difference in any parameters related to male sexual behaviors in Foxp2^mCherry^ and Foxp2^hM4Di^ male mice. **(b)** Friedman tests followed by multiple comparison tests with FDR correction; p < 0.0001. **(c, e, g-l, n-s)** Two-way repeated measures ANOVA followed by Sidak’s multiple comparison tests; **(d)** Two-tailed McNemar’s test. n = number of animals. Data are mean ± SEM. **p < 0.01, ***p < 0.001. Otherwise, p > 0.05. See Source Data Extended Data Fig. 6 for more detailed statistics. Source data

To understand the functional importance of MeA^Foxp2^ and MeA^Dbx1^ cells in social behaviors, we bilaterally injected Cre-dependent and Flp-dependent hM3Dq viruses into the MeA of *Foxp2*^*cre*^ and *Dbx1*^*cre*^*;LSL-FlpO* naive male mice, respectively (Foxp2^hM3Dq^ and Dbx1^hM3Dq^) (Fig. 7a,b). Control animals were injected with mCherry virus in the MeA (Foxp2^mCherry^ and Dbx1^mCherry^). Three weeks later, we intraperitoneally (i.p.) injected saline and clozapine-*N*-oxide (CNO) on two consecutive days and, 30 min later, introduced a pup, an adult male and a female intruder into the cage sequentially, each for 5–10 min with 5 min in between (Fig. 7c). To determine whether MeA^Foxp2^ activation could result in increases in aggression in mice that are not spontaneously aggressive, we first tested animals’ baseline aggression level after saline injection on day 1, followed by CNO injection on day 2. Although only four of 10 Foxp2^hM3Dq^ male mice attacked a male intruder after saline injection, all Foxp2^hM3Dq^ males attacked the intruder after CNO injection (Fig. 7d,e). In comparison, only four of eight control Foxp2^mCherry^ mice initiated attack after CNO injection (Fig. 7d,e). The total attack time of Foxp2^hM3Dq^ males significantly increased after CNO injection (Fig. 7f), although the latency to attack did not decrease in animals that attacked on both days (Extended Data Fig. 6a). Possibly due to increased aggression, Foxp2^hM3Dq^ mice spent less time investigating the male intruder after CNO injection (Fig. 7g). For most Foxp2^hM3Dq^ animals (9/10), we tested the aggression level again on day 3 after saline injection and observed a significantly lower total attack duration in comparison to that of day 2 after CNO injection, further suggesting that the longer attack duration after Foxp2^hM3Dq^ activation is not simply due to a natural increase in aggression with repeated resident–intruder (R–I) tests (Extended Data Fig. 6b). Furthermore, CNO-induced attack was not due to an increase in general arousal, as locomotion did not differ between saline-injected and CNO-injected days (Extended Data Fig. 6c). Notably, the increased aggression is adult male specific, as we did not observe an increase in infanticide after activating MEA^Foxp2^ cells (Extended Data Fig. 6d). The overall pup interaction was also unchanged (Extended Data Fig. 6e). Similarly, male sexual behaviors, including female investigation, mounting and intromission, were not affected by MEA^Foxp2^ activation (Extended Data Fig. 6f–l). Control Foxp2^mCherry^ animals showed no significant change in any social behavior after CNO injection compared to saline injection (Fig. 7d–g and Extended Data Fig. 6d–l).

**Extended Data Fig. 7:**
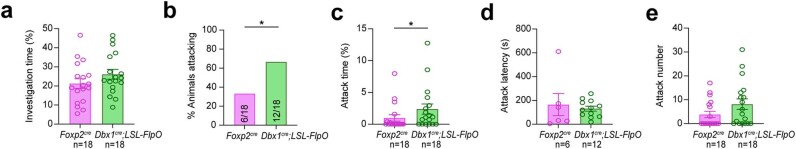
The aggression level of *Foxp2*^*cre*^ and *Dbx1*^*cre*^*;LSL-FlpO* naïve male mice, related to Fig. 7. **(a)** Percent of time *Foxp2*^*cre*^ and *Dbx1*^*cre*^*;LSL-FlpO* male mice spent investigating a male intruder. **(b)** Percent of *Foxp2*^*cre*^ and *Dbx1*^*cre*^*;LSL-FlpO* male mice that attacked a male intruder. **(c)** Percent of time *Foxp2*^*cre*^ and *Dbx1*^*cre*^*;LSL-FlpO* male mice spent attacking a male intruder. **(d)** Attack latency of *Foxp2*^*cre*^ and *Dbx1*^*cre*^*;LSL-FlpO* male mice that attacked the male intruder. **(e)** Number of attacks of *Foxp2*^*cre*^ and *Dbx1*^*cre*^*;LSL-FlpO* male mice towards a male intruder. Data are the same as those in Fig. 7e–k after day 1 saline injection**. (a,d)** Two-tailed unpaired t-test. **(b)** Two-tailed Chi-Square test; p = 0.0455 **(c,e)** Two-tailed Mann-Whitney test; **(c)** p = 0.0465. n = number of animals. Data are mean ± SEM; *p < 0.05. Otherwise, p > 0.05. See Source Data Extended Data Fig. 7 for more detailed statistics. Source data

**Extended Data Fig. 8:**
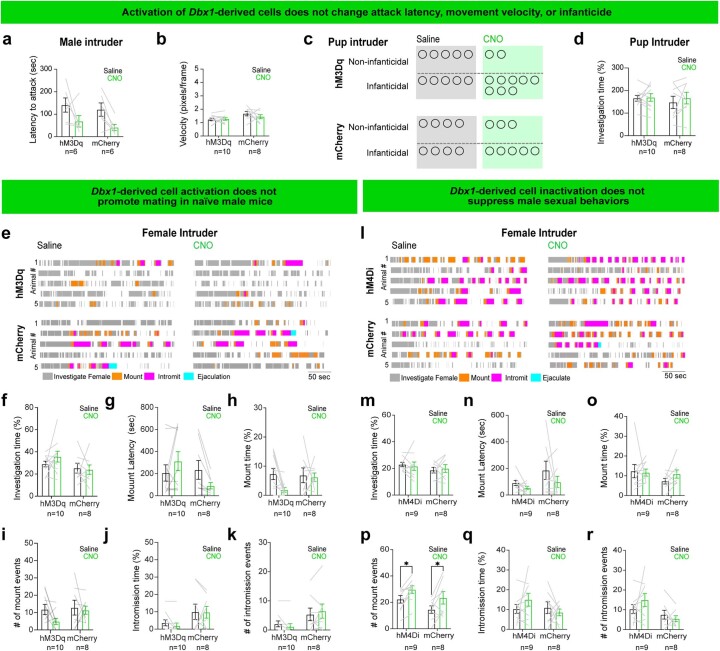
Additional behavioral assays during chemogenetic activation and inactivation of MeA^Dbx1^ cells, related to Fig. 7. **(a)** In Dbx1^hM3Dq^ and Dbx1^mCherry^ male mice, the latency to attack a male intruder after CNO injection did not differ from that after saline injection. Only animals that showed attack after both saline and CNO injections were included in this analysis. **(b)** Velocity (pixels/frame) of Dbx1^hM3Dq^ or Dbx1^mCherry^ male mice in a 5 min period after 30 min CNO or saline injection when the test animal was alone in its home cage. **(c)** Number of Dbx1^hM3Dq^ and Dbx1^mCherry^ male mice that attacked pups vs. those that did not after saline or CNO injection. Each circle represents one mouse. **(d)** Percentage of time Dbx1^hM3Dq^ and Dbx1^mCherry^ male mice spent investigating the pup after saline or CNO injection. **(e)** Representative raster plots showing the behaviors towards an adult female intruder of 5 Dbx1^hM3Dq^ and 5 Dbx1^mCherry^ mice after i.p. injection of saline or CNO. **(f-k)** No difference in male sexual behaviors after CNO injection in comparison to saline injection in Dbx1^mCherry^ or Dbx1^hM3Dq^ male mice. **(l)** Representative raster plots showing the behaviors towards an adult female intruder of 5 Dbx1^hM4Di^ and 5 Dbx1^mCherry^ mice after i.p. injection of saline or CNO. **(m-r)** No difference in male sexual behaviors after CNO injection in comparison to saline injection in Dbx1^mCherry^ or Dbx1^hM4Di^ male mice. **(a, b, d, f-k, m-r)** Two-way repeated measures ANOVA followed by Sidak’s multiple comparison tests. **(c)** Two-tailed McNemar’s test. n = number of animals. Data are mean ± SEM. All p > 0.05. See Source Data Extended Data Fig. 8 for more detailed statistics. Source data

We noticed that *Dbx1*^*cre*^*;LSL-FlpO* male mice tend to be more aggressive than *Foxp2*^*cre*^ male mice, possibly due to their difference in genetic background (Extended Data Fig. 7)^26,27^. Twelve of 18 *Dbx1*^*cre*^*;LSL-FlpO* animals (combining mCherry and hM3Dq groups) attacked the intruder during the first encounter (after saline injection), whereas only six of 18 *Foxp2*^*cre*^ animals did so (Extended Data Fig. 7b). Notably, there was no difference between Dbx1^hM3Dq^ and Dbx1^mCherry^ groups in the percentage of animals that attacked (Fig. 7i). The latency to attack and attack duration also did not differ on CNO-injected and saline-injected days in both Dbx1^hM3Dq^ and Dbx1^mCherry^ groups (Fig. 7j and Extended Data Fig. 8a), although Dbx1^hM3Dq^ male mice investigated the male intruder less after CNO than saline injection (Fig. 7k). Activating MeA^Dbx1^ cells did not change the probability of infanticide, male sexual behaviors or locomotion significantly (Extended Data Fig. 8b–k). Thus, MEA^Foxp2^ cells can specifically drive inter-male aggression in even non-aggressive naive male mice, whereas activating MEA^Dbx1^ cells does not promote any specific social behaviors to a significant level.

### Inhibiting MeA^Foxp2^ cells reduces aggression in experienced animals

We next asked whether MeA^Foxp2^ and MeA^Dbx1^ cells are necessary for social behaviors, including inter-male aggression. We injected Cre-dependent and Flp-dependent hM4Di-mCherry into the MeA of *Foxp2*^*cre*^ and *Dbx1*^*cre*^*;LSL-FlpO* male mice, respectively (Foxp2^hM4Di^ and Dbx1^hM4Di^). Control animals were injected with mCherry virus (Fig. 7l,m). Three weeks after viral injection, all animals went through repeated R–I tests until they showed stable aggression (Fig. 7n). Then, we i.p. injected saline and CNO on separate days in a randomized order and, 30 min later, tested the behaviors against a male and a receptive female intruder, each for 10 min (Fig. 7n). After CNO injection, Foxp2^hM4Di^ mice spent more time investigating and less time attacking the male intruder (Fig. 7o–q). The latency to attack increased significantly (Fig. 7r). Foxp2^mCherry^ mice showed no difference in male investigation or attack duration between CNO-injected and saline-injected days (Fig. 7o–r). In contrast, CNO injection in Dbx1^hM4Di^ mice did not result in significant changes in male investigation, aggressive behaviors or latency to attack (Fig. 7s–v). CNO injection in Foxp2^hM4Di^ or Dbx1^hM4Di^ mice caused no change in female investigation or any aspects of male sexual behaviors except an increase in mount number in both Dbx1^hM4Di^ and Dbx1^mcherry^ groups (Extended Data Figs. 7m–s and 8l–r). These results suggest that MeA^Foxp2^ cells specifically modulate inter-male aggression, whereas MeA^Dbx1^ cells do not.

### Differential outputs of MeA^Dbx1^ and MeA^Foxp2^ cells

**Fig. 8:**
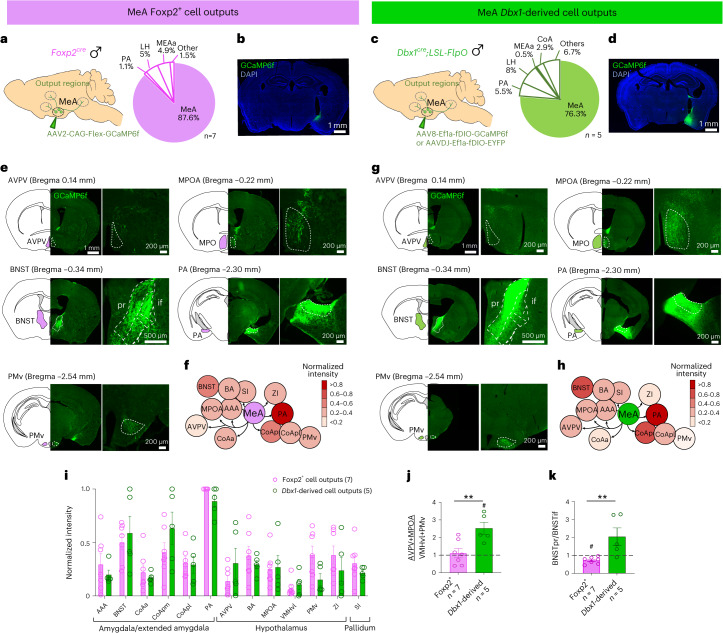
Outputs of MeA^Foxp2^ and MeA^Dbx1^ cells. **a**,**c**, Strategies for anterograde viral tracing of MeA^Foxp2^ (**a**) and MeA^Dbx1^ (**c**) cells. Pie charts showing the distribution of infected cells. **b**,**d**, Representative histological images showing the infected cells in *Foxp2*^*cre*^ (**b**) and *Dbx1*^*cre*^*;LSL-FlpO* (**d**) mice. Green: GCaMP6f expression. Blue: DAPI staining. **e**,**g**, Representative histological images showing MeA^Foxp2^ (**e**) and MeA^Dbx1^ (**g**) projections at various downstream regions. The gain of PA and BNST images in **g** was reduced to avoid complete saturation. **f**,**h**, Overviews of MeA^Foxp2^ (**f**) and MeA^Dbx1^ (**h**) cell outputs. **i**, The intensity of MeA^Foxp2^ and MeA^Dbx1^ projection fields in various regions, calculated as the average pixel intensity in a given region divided by the maximum mean intensity value among all regions with ≥0.2 normalized intensity and the VMHvl. **j**, The intensity of fibers, originating from MeA^Foxp2^ and MeA^Dbx1^ cells, at AVPV and MPOA (anterior medial hypothalamic regions) over the VMHvl and PMv (posterior medial hypothalamic regions). The dotted line denotes *y* = 1. **k**, The intensity of fibers, originating from MeA^Foxp2^ and MeA^Dbx1^ cells, at BNSTpr over that in BNSTif. The dotted line denotes *y* = 1. **i**, Two-way repeated-measures ANOVA followed by Sidak’s multiple comparison tests. **j**,**k**, Two-tailed unpaired *t*-test; *P* = 0.0081 (**j**) and *P* = 0.0095 (**k**). Parentheses and *n* indicate number of animals. All statistical tests are two-tailed. Data are mean ± s.e.m. **P < 0.01, otherwise *P* > 0.05. One-sample *t*-test for each behavior with null hypothesis ratio = 1, corrected for repeated testing with FDR of 0.05. #*q* < 0.05, otherwise *q* > 0.05. See Extended Data Table 1 for brain region abbreviations. See Source Data Fig. 8 for more detailed statistics. Source data

**Extended Data Fig. 9:**
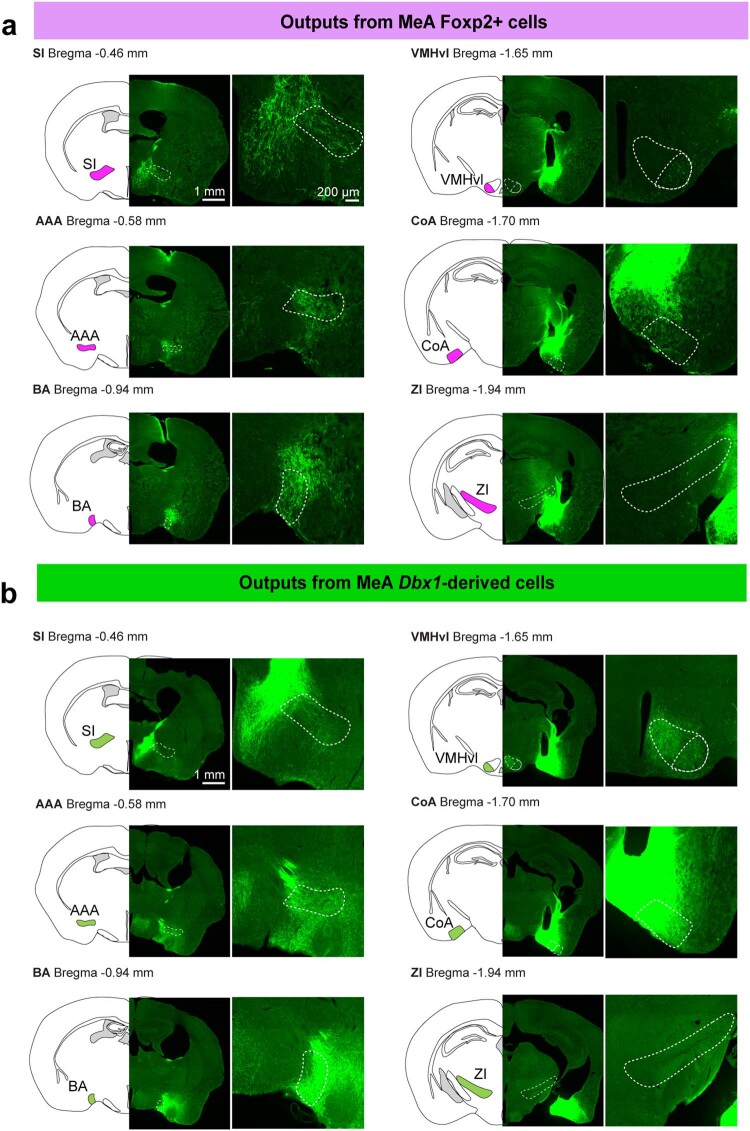
Brain regions downstream of MeA^Foxp2^ and MeA^Dbx1^ cells, related to Fig. 8. **(a-b)** Representative images of 6 brain regions showing the GFP fibers originating from MeA^Foxp2^ **(a)** and MeA^Dbx1^ **(b)** cells.

As MeA^Dbx1^ and MeA^Foxp2^ cells play distinct roles in driving social behaviors, presumably through their differential connections on downstream cells, we next asked whether these two MeA subpopulations differ in their projections using anterograde virus tracing (Fig. 8a–d). We observed that both MeA subpopulations project mainly to other extended amygdala areas, such as PA, CoA, posterior BNST (BNSTp) and medial hypothalamus (MH) (Fig. 8e–i and Extended Data Fig. 9). The average density of projections originating from MeA^Dbx1^ and MeA^Foxp2^ did not differ significantly in any specific amygdala or hypothalamic nucleus (Fig. 8i). Nevertheless, we observed some differences in projection patterns. The MeA^Dbx1^ cells generally provide more inputs to AVPV and MPOA subnuclei, structures related to sexual behaviors^33,34^, than the VMHvl and PMv, structures related to aggression^3^. The overall fiber density from MeA^Dbx1^ cells in MPOA + AVPV is approximately twice that in VMHvl + PMv (Fig. 8i, j). In contrast, the density of the fibers originating from MeA^Foxp2^ cells is similar in the sexual-behavior-related and aggression-related medial hypothalamic regions (Fig. 8i,j). Furthermore, a close analysis of the BNST revealed differential patterns of projections from MeA^Dbx1^ and MeA^Foxp2^ cells. Whereas MeA^Dbx1^ cells target the principal nucleus of the BNST (BNSTpr) primarily, MeA^Foxp2^ cells project more densely to the interfascicular part of the BNST (BNSTif) (Fig. 8k).

## Discussion

In this study, we showed that two MeA subpopulations, marked by the expression of different transcription factors from early development, play distinct roles in social behaviors, show differential input and output patterns and are responsive to different conspecific sensory cues. The male-specific responses of MeA^Foxp2^ cells exist before puberty and aggression onset, suggesting that these responses are largely developmentally hardwired. The reliability and temporal precision of MeA^Foxp2^ cell responses improve with adult social experience, demonstrating distinct roles of nature versus nurture in setting up the response patterns of this population.

### MeA^Foxp2^ and MeA^Dbx1^ cell activity and function in social behaviors

Our previous work identified two developmentally distinct GABAergic MeA subpopulations marked by the expression of Dbx1 and Foxp2 (refs. ^9,23^). They differ in sex steroid hormone receptor expression, ion channel composition and intrinsic electrophysiological properties^9,35^. Our current study further revealed their distinct functions in social behaviors that are well matched with their differential connectivity and in vivo response patterns. These results suggest that social circuits at the MeA could be largely hardwired according to transcription-factor-defined genetic programs.

MeA^Foxp2^ cells responded strongly during both male investigation and attack. Functionally, chemogenetic activation of MeA^Foxp2^ cells promoted attack even in non-aggressive male mice. Anatomically, MeA^Foxp2^ cells receive very little direct input from the AOB. Thus, despite the long-recognized role of the MeA in pheromone processing^6,7,10,36,37^, MeA^Foxp2^ cells appear to be more involved in facilitating aggressive actions. However, our population fiber photometry recording does not provide single-cell resolution. Therefore, it remains possible that distinct subsets of MeA^Foxp2^ cells process olfactory cues and mediate aggressive actions.

In contrast to MeA^Foxp2^ cells, MeA^Dbx1^ cells are tuned to broad social cues, including those from males, females and pups and respond robustly during ejaculation but minimally during attack or other copulatory behaviors. Consistent with the lack of activity increase during attack, inactivation of MeA^Dbx1^ cells does not impair male aggression, and chemogenetic activation of MeA^Dbx1^ cells does not promote attack. Given that MeA^Dbx1^ cells are three times more abundant than MeA^Foxp2^ cells and more excitable^9^, yet fail to promote attack, we conclude that aggression generation requires activation of a specific transcriptionally defined subpopulation instead of any subset of MeA GABAergic cells.

Given the response pattern of MeA^Dbx1^ cells, we considered their primary role in processing social cues during the investigatory phase. However, animals with inactivated MeA^Dbx1^ cells properly directed their attack toward males and mount toward females, suggesting that MeA^Dbx1^ cells are dispensable for sex discrimination. This negative result is possibly due to the existence of other extended amygdala populations that can readily distinguish male and female cues^38^—for example, MeA^Foxp2^ and aromatase cells in BNSTpr^39^. Interestingly, we found that MeA^Dbx1^ cells robustly increase activity during male ejaculation. Recent works found that Esr2^+^ cells in the BNST are also highly activated during ejaculation and functionally important for diminished sexual motivation after ejaculation^38,40^. Here, we observed a tendency of decreased mounting and intromission duration after activating MeA^Dbx1^ cells (Extended Data Fig. 6p,r), hinting at the possible role of MeA^Dbx1^ cells in suppressing sexual motivation as BNST^Esr2^ cells. This hypothesis, however, requires further investigation in future studies.

Previous work showed that MeA GABAergic cells are activated during pup-directed attacks and can promote infanticide^22^. However, neither MeA^Foxp2^ nor MeA^Dbx1^ cells increased activity during pup-directed aggression or affected infanticide when artificially activated, suggesting that MeA^Foxp2^ cells are specialized for adult-directed aggression. Therefore, other GABAergic subclasses likely exist for driving infanticide and remain to be identified.

### Developmentally wired versus experientially wired

There is an ongoing debate regarding whether the responses of cells in the SBN are developmentally hardwired or established through adult social experience. In the VMHvl, an essential region for male aggression^41–43^, individual cell responses to male and female cues overlap extensively in naive adult male mice and diverge only after repeated interaction with females^44^. In contrast, aromatase-expressing cells in male BNSTpr preferentially respond to female cues over male cues, even in naive animals^39^. Ca^2+^ imaging in the MeA revealed that approximately half of MeA cells are tuned to one stimulus in naive animals, and, after sexual experience, the proportion of cells that are responsive to the opposite sex increases, denoting experience-dependent activity refinement^10^. In our study, MeA^Foxp2^ cells showed strong male-biased responses in naive animals, suggesting that male olfactory inputs are developmentally wired to target MeA^Foxp2^ cells. However, the responses of MeA^Foxp2^ cells in naive males are slow and inconsistent and only become fast and consistent after repeated social interactions and aggressive encounters, suggesting that adult social experience plays an important role in refining this hardwired circuit to improve its input (sensory cue)–output (spiking) transformation efficiency.

How is the male-specific response of MeA^Foxp2^ cells achieved during development? The classical ‘organization/activation’ model states that gonadal hormones act in two phases to establish sex-specific circuits^45–47^. First, during the organization stage, gonadal hormones during prenatal development set up the basic structure and connection of the circuit. Then, the circuits are activated by gonadal hormones during puberty to generate appropriate sex-specific social behaviors. In male mice, puberty occurs between P30 and P38 when testosterone spikes and aggression emerges^28,45^. The fact that male-biased responses of MeA^Foxp2^ cells exist before puberty suggests that male cues have been wired preferentially to MeA^Foxp2^ cells during the organization stage. After puberty, MeA^Foxp2^ cells show enhanced male-biased responses due to decreased responses to non-male social cues, suggesting further circuit refinement possible through local inhibition. Altogether, we propose that the response specificity of MeA^Foxp2^ cells during development is achieved through a multi-stage process, including pre-pubertal hardwiring, pubertal refinement and adult social experience-dependent potentiation. Future microcircuit studies could help further validate this model and its generality in the SBN.

### Social behavior circuits beyond MeA

In mice, olfactory inputs are the most essential for determining the identity of a conspecific—for example, its sex, age, social ranking and health state (for example, sickness)^48^. Because MeA^Foxp2^ cells receive little direct input from the AOB and other primary olfactory relays, we speculate that MeA^Foxp2^ cells obtain highly ‘processed’ olfactory information from the PA. Recent works revealed that PA cells projecting to the VMHvl are crucial for territorial aggression, and these cells are activated during both male investigation and attack^49,50^. The PA also projects strongly to MeA; however, whether this projection is essential for aggression remains to be explored. On the contrary, MeA^Dbx1^ cells receive abundant inputs from AOB and other primary olfactory processing regions, which could be responsible for the broad and fast responses of MeA^Dbx1^ cells to various social cues.

At the output level, MeA^Dbx1^ and MeA^Foxp2^ cells project to distinct pBNST subnuclei: MeA^Dbx1^ primarily to the BNSTpr and MeA^Foxp2^ cells mainly to the BNSTif. Miller et al.^19^ recently demonstrated that MeA cells that express D1R target the BNSTif primarily, and activating MeA^D1R^-BNST projections increased territorial aggression. This supports the relevant role of BNSTif in aggression and a potential downstream mechanism by which MeA^Foxp2^ cells mediate aggressive action. Additionally, MeA^Foxp2^ cells project relatively more densely to the VMHvl and PMv than MeA^Dbx1^ cells. Given that VMHvl and PMv are central for male aggression^42,43,51^, the more robust projection of MeA^Foxp2^ cells to these regions is consistent with the essential role of MeA^Foxp2^ cells in male aggression.

Our study identified a developmentally hardwired circuit at the MeA to process male information essential for initiating aggression. We revealed the distinct contribution of development versus experience in social information processing and highlighted a lineage-based organization strategy that enables the same SBN to drive diverse social behaviors^2^.

## Methods

### Mice

All animal procedures were approved by the Institutional Animal Care and Use Committee of NYU Langone Health under protocol IA16-01416. Adult and juvenile experimental and stimulus mice were housed under a 12-h light/dark cycle (10:00 to 22:00 dark) with water and food ad libitum. Holding and experimental room temperatures were maintained at 20–22 °C and humidity kept between 30% and 70% (average ~45%). After surgical procedures, all experimental animals were single housed. The *Foxp2*^*cre*^ mice were originally provided by Richard Palmiter (now Jackson Laboratory stock no. 030541)^26^. The *Dbx1*^*cre*^ mice were originally provided by Alessandra Pierani and crossed to the Flp-excised and Cre-inducible *LSL-FlpO* mouse line or to the Ai6 mouse line (Jackson Laboratory stock no. 028584 and no. 007906, respectively)^24,25,27^. Both *Foxp2*^*cre*^ and *Dbx1*^*cre*^ mice are black, whereas the fur color of *LSL-FlpO* mice is agouti. Stimulus animals were C57BL/6N and 129S4/SvJae group-housed females, pups (P1–P7) and group-housed BALB/c males purchased from Charles River Laboratories and bred in-house. Females were considered receptive if an experienced male could mount and intromit the female in at least three attempts. No statistical methods were used to pre-determine sample sizes, but our sample sizes are similar to those reported in previous publications^49,52,53^.

### Viruses and stereotaxic surgery

For fiber photometry experiments, we injected 100 nl of AAV2-CAG-Flex-GCaMP6f (2.21 × 10^13^ vector genomes per milliliter (vg/ml) or 1.82 × 10^12^ vg/ml; UPenn Viral Core) unilaterally into the MeA (AP = −1.5 mm, ML = 2.15 mm, DV = −5.1 mm) of *Foxp2*^*cre*^ male mice. For *Dbx1*^*cre*^*;LSL-FlpO* mice, we injected either 100 nl of AAV8-Ef1a-fDIO-GCaMP6f (1 × 10^13^ vg/ml; kindly provided by Naoshige Uchida) or 120 nl of mixed AAV9-Ef1a-fDIO-Cre (2.5 × 10^13^ vg/ml; Addgene) and AAV2-CAG-Flex-GCaMP6f (1:2; 2.21 × 10^13^ vg/ml; UPenn Viral Core) or 150 nl of AAV2-Ef1a-fDIO-GCaMP6f (4.1 × 10^12^ vg/ml; UNC Vector Core) into the MeA. For fiber photometry recordings in *Foxp2*^*cre*^ juvenile mice, we injected 100 nl of AAV1-CAG-Flex-GCaMP6f (9.4 × 10^12^ vg/ml; UPenn Viral Core) unilaterally into the developing MeA (AP = −0.7 mm, ML = 2.03 mm, DV = −5.05 mm). For chemogenetic experiments, we bilaterally injected 400–600 nl of AAV1-Ef1a-DIO-hM4D(Gi)-mCherry, 150 nl of AAV2-hSyn-DIO-hM3D(Gq)-mCherry or 150–600 nl of AAV2-hSyn-DIO-mCherry (3 × 10^12^ vg/ml, 5.1 × 10^12^ vg/ml and 5.6 × 10^12^ vg/ml, respectively; Addgene and UNC Vector Core) into the MeA of *Foxp2*^*cre*^ mice. For chemogenetic experiments in *Dbx1*^*cre*^*;LSL-FlpO* mice, we injected 300 nl of AAVDJ-hSyn-fDIO-hM4D(Gi)-mCherry, 50–60 nl of AAV2-Ef1a-fDIO-hM3D(Gq)-mCherry (Vigene) and 60–120 nl of AAV2-Ef1a-fDIO-mCherry (2.65 × 10^13^ vg/ml, 1.84 × 10^13^ vg/ml and 1.1 × 10^13^ vg/ml, respectively; Addgene). For monosynaptic retrograde rabies experiments in *Foxp2*^*cre*^ mice, we injected unilaterally into the MeA 200–250 nl of mixed AAV1-CA-Flex-RG and AAV8-Ef1-Flex-TVA-mCherry (1:1; 3 × 10^12^ vg/ml and 5.4 × 10^12^ vg/ml; UNC Vector Core) and, 4 weeks later, 800 nl of EnvA G-Deleted Rabies-eGFP (2.26 × 10^8^ and 1.07 × 10^8^ transforming units per milliliter (TU/ml); Salk Viral Vector Core). For monosynaptic retrograde rabies experiments in *Dbx1*^*cre*^*;LSL-FlpO* mice, we injected mixed 110–120 nl of AAV8-Flex(FRT)-G and AAV8-Flex(FRT)-TVA-mCherry (1:1; 1.82 × 10^13^ vg/ml and 1.39 × 10^13^ vg/ml; Stanford Gene Vector and Viral Core) and, 4 weeks later, 800 nl of EnvA G-Deleted Rabies-eGFP (Salk Viral Core). We also unilaterally injected 80–100 nl of AAVDJ-Ef1a-fDIO-EYFP (2.1 × 10^12^ vg/ml; UNC Vector Core) into the MeA of *Dbx1*^*cre*^*;LSL-FlpO* mice for anterograde tracing experiments. For Chr2-assisted circuit mapping, we injected 150 nl of AAV2-Flex-GFP (3.7 × 10^12^ vg/ml; UNC Vector Core) unilaterally into the MeA of *Foxp2*^*cr*e^ mice and 40–200 nl of AAV9-hSyn-ChrimsonR-tdTomato (5.5 × 10^12^ vg/ml; Addgene) unilaterally into the olfactory bulb (AP = 4.45 mm, ML = 0.25 mm, DV = −1.55 mm) of *Foxp2*^*cre*^ and *Dbx1*^*cre*^*;Ai6*^*+/−*^ mice. EnvA G-deleted Rabies virus titers were 2.26 × 10^8^ and 1.07 × 10^8^ TU/ml.

During surgery, adult male mice were anesthetized with isoflurane (2%) and then placed in a stereotaxic apparatus (Kopf Instruments). For fiber photometry recordings in juvenile mice, P11 pups were anesthetized with isoflurane (2%) and placed in a stereotaxic apparatus modified with a neonatal anesthesia head holder and zygoma ear cups (Kopf Instruments). The virus was then delivered into the target region of interest through a glass capillary using a nanoinjector (World Precision Instruments). For fiber photometry experiments in adults, a 400-μm fiber optic cannulae with ceramic ferrule (Thorlabs, FT400EMT, CF440-10 or RWD, R-FOC-L400C-50NA) was placed 0.3 mm dorsal to the viral injection site and cemented with adhesive dental cement (C&B Metabond, S380). For juvenile experiments, juveniles at P24 were implanted with the optical fiber in the MeA (AP = −0.7 mm, ML = 2.03 mm, DV = −4.75 mm). Histology analysis was performed for all animals, and only animals with correct virus expression and fiber placement were used for the final analysis.

### Behavioral assays and analysis

Behavior was recorded by two synchronized top and side cameras (Basler, acA640-100gm) at 25 frames per second and digital video recording software (StreamPix 5, NorPix) in a dark room with infrared lights. Behaviors were manually annotated on a frame-by-frame basis by using a custom MATLAB function called ‘BehaviorAnnotator’ (https://github.com/pdollar/toolbox).

For male–male interactions, we annotated investigation, groom and attack. For male–female interactions, we recorded investigation, mount, intromission and ejaculation. For male–pup interactions, we recorded investigation, groom and infanticide. For fiber photometry analysis, investigation and groom were combined as ‘Investigation’. ‘Investigation’ was considered nose contact with any body part of the target mouse. ‘Groom’ was classified as when a mouse has its front paws holding the back or face of the target mouse or pup and licking either face or back. ‘Attack’ was determined as a series of actions by which the male mouse lunges, bites, chases and pushes the target mouse. ‘Mount’ was defined as a series of fast movements by which the male mouse places its front paws on the target mouse and positions itself on top of the target mouse. ‘Intromission’ was annotated as deep rhythmic thrusts following the mount. ‘Ejaculation’ was considered when the male stops deep thrusting and freezes in place for several seconds while firmly holding the female mouse and then slumping to the side. ‘Infanticidal behavior’ was considered as biting the pup that results in tissue damage. For fiber photometry and chemogenetic analysis, pup investigation and groom were combined as ‘pup investigation’.

In this study, experiments were not performed in a blinded manner as the experimental conditions were clear to the experimenters and the analyses were carried out using a recording system not subject to human bias. During behavioral annotations, the experimenter was blinded to the GCaMP6 signal or to the behavioral response.

### Fiber photometry

*Foxp2*^*cre*^ and *Dbx1*^*cre*^*;LSL-FlpO* randomly selected male mice aged 2–8 months were used for adult fiber photometry recordings. *Foxp2*^*cre*^ male mice starting at age P25 were used for juvenile fiber photometry experiments. For adult head-fixed experiments, the mice were naive and did not have any interactions with other conspecifics outside of their littermates and dams. The recording mouse was head-fixed using a 3D-printed head ring and placed on a 3D-printed wheel^54^ for a minimum of 3 d of training before testing. Each stimulus was presented five times for 10 s with a 40-s interval in between presentations and a minimum of 5-min break in between different stimuli. Male and receptive female stimulus mice were anesthetized with ketamine (100 mg kg^−1^) and xylazine (10 mg kg^−1^).

Fiber photometry was performed as previously described ^49,55,56^. Fiber photometry signals were collected by using a custom Tucker-Davis Technologies (TDT) program, OpenEx. To analyze changes in Ca^2+^ activity, the MATLAB function ‘msbackadj’, with a moving window of 25% of the total recording time, was used to obtain the instantaneous baseline signal (F_baseline_). The instantaneous ΔF/F was calculated as (F_raw_ − F_baseline_) / F_baseline_. The *z*-score of the ΔF/F (Fz) was obtained using the MATLAB function ‘zscore’ for the whole trace. The peri-event histograms (PETHs) were calculated by aligning the Fz of each trial to either the onset or offset of each behavior. In recordings of head-fixed naive male mice (Fig. 2), the Fz peak was calculated by obtaining the average of the maximum value during stimulus presentation. In recordings of freely moving animals, the introduction Fz peak was calculated by obtaining the maximum value during the first 100 s of stimulus introduction into the resident’s cage. The Fz mean was calculated by obtaining the average of the mean values during specific behaviors during the specified intruder presentation window. The male PI was calculated as (Z_investigate male_ − 0.5 × (Z_investigate female_ + Z_investigate pup_)) / (|Z_investigate male_| + 0.5 × |Z_investigate female_ + Z_investigate pup_|); the female PI was calculated as (Z_investigate female_ − 0.5 × (Z_investigate male_ + Z_investigate pup_)) / (|Z_investigate female_| + 0.5 × |Z_investigate male_ + Z_investigate pup_|); and the pup PI was calculated as (Z_investigate pup_ − 0.5 × (Z_investigate male_ + Z_investigate female_)) / (|Z_investigate pup_| + 0.5 × |Z_investigate female_ + Z_investigate male_|).

When recording from freely moving mice (Figs. 3–5), a receptive female and an adult male mouse were introduced into the cage for 10 min. A pup was placed in the cage for 5 min. The male intruder was placed in the cage for >10 min until the recording mice elicited more than six attacks, without exceeding 1 h in the cage. A receptive female was introduced until 5 min after the recording mouse ejaculated.

When comparing naive freely moving and experienced male mice responses, the ‘latency to respond’ was calculated as the time lapse from behavior onset to when the response reaches Z ≥ 1. The ‘% of trials respond’ was calculated as the percentage of trials that reached Z ≥ 1. ‘Investigation time per trial (s)’ was calculated as the average duration of all male investigation trials. Heat maps were constructed as F_Z_ − Fz at time 0 for each trial.

### Chemogenetic-mediated activation and silencing

For chemogenetic activation experiments, randomly selected naive male mice with no prior social experience except with their dam and littermates were used. On day 1, male mice were i.p. injected with saline. Thirty minutes after injection, video recordings started. After a 5-min baseline period, a pup intruder was placed into the cage for 5 min, followed by a 10-min presentation of an adult male and a receptive female, with 5-min breaks between stimulus. On day 2, male mice were i.p. injected with 1 mg kg^−1^ of CNO (Sigma-Aldrich, C0832), and stimuli presentation was repeated as on day 1. On day 3, *Foxp2*^*cre*^ male mice were i.p. injected with saline, and stimuli were introduced as on days 1 and 2.

For chemogenetic silencing experiments, experimental male mice were trained to attack by introducing an adult male mouse daily for 10–30 min per day until they could consistently attack within a 10-min period. Mice were then i.p. injected with saline or CNO (1 mg kg^−1^) on interleaved days for one or two rounds. Thirty minutes after injection, behavioral recordings started, and, after a 5-min baseline period, an adult male or a receptive female was introduced into the cage for 10 min each, with a 5-min break.

### Animal body tracking

The velocity (pixels per frame) of each animal after 30 min of saline or CNO i.p. injection was obtained during the first 5 min of the chemogenetic assay before the introduction of any stimulus. The location of each animal was tracked using the top-view camera recordings and analyzed using a custom-written MATLAB GUI and code (https://github.com/pdollar/toolbox)^42^.

### Distribution of MeA^Foxp2^ and MeA^Dbx1^ cells across the medial-lateral and dorsal-ventral axis

MeApd^Foxp2^ and MeApd^Dbx1^ cells were counted using Adobe Photoshop. The optic tract was used to calculate the medial-lateral cell distribution, whereas the dorsal edge of the MeAp was used to calculate the dorsal-ventral cell distribution. The ‘point to distance’ MATLAB function was used to calculate the distance from a point (that is, MeA^Foxp2^ or MeA^Dbx1^ cells) to a line (that is, the optic tract or the dorsal edge of the MeApd). Wilcoxon matched-pairs rank tests were performed to compare the distance distributions of MeApd^Foxp2^ and MeApd^Dbx1^ cells.

### Triple in situ hybridization RNAscope

For in situ hybridization, brains were perfused with 1× PBS and fresh frozen in dry ice. Brains were embedded in O.C.T. compound (Sakura, 4583) and cut in 20-µm sections using a cryostat (Leica, CM1950) and placed directly into slides (Fisherbrand Superfrost Plus microscope slides, Thermo Fisher Scientific, 22-037-246). Every sixth section containing the MeA (bregma −1.4 mm to −2.1 mm) was used for staining. Using the manufacturer’s protocol (Advanced Cell Diagnostics)^57^, slides containing the sections were dehydrated with several steps of ethanol and digested with proteinase, followed by hybridization of the mixed target probes for *GFP* (538851-C2), *Slc32a1* (319191) and *Slc17a6* (319171-C3). Slides were then stained with DAPI and coverslipped. Sections were imaged using a confocal microscope (Zeiss, LSM 800).

### Immunohistochemistry and imaging analysis

Mice were anesthetized and perfused with 1× PBS, followed by 20 ml of 4% paraformaldehyde (PFA). Brains were fixed in 4% PFA for 6–12 h at 4 °C, dehydrated in 15% sucrose overnight, embedded in O.C.T. compound (Sakura, 4583) and cut into 50-µm sections using a cryostat (Leica, CM1950). Every third section was used for immunohistochemistry performed as previously described^9^. In brief, free-floating sections were incubated with primary antibody and blocked in 10% normal donkey serum (Jackson ImmunoResearch, 017-000-121) overnight. Brain sections were washed, placed in secondary antibody and blocked for 4–16 h. Then, sections were washed, mounted (Thermo Fisher Scientific, 12-550-15) and coverslipped using Fluoromount mounting media with DAPI (Thermo Fisher Scientific, 00-4959-52). Primary antibodies used were rabbit anti-Foxp2 (1:500, Abcam, ab16046), rat anti-GFP (1:1,000, Nacalai, 04404-84) and rabbit anti-mCherry (1:1,000, TaKaRa, Living Colors DsRed Polyclonal Ab 632496). Secondary antisera used were donkey anti-rat Alexa Fluor 488 (1:300; Jackson ImmunoResearch, 712-545-150) and donkey anti-rabbit Cy3 (1:1,000, Jackson ImmunoResearch, 711-165-152). Sections were imaged using a slide scanner (Olympus, VS120) or a confocal microscope (Zeiss, LSM 800) and counted manually using Adobe Photoshop. Cells stained with DAPI were automatically counted using the ‘analyze particles’ feature in ImageJ software and manually corrected.

### Monosynaptic retrograde rabies input mapping

To determine the inputs to MeA^Foxp2^ and MeA^Dbx1^ cells, we injected randomly selected adult male mice with Cre-dependent or Flp-dependent AAV-G and AAV-TVA-mCherry viruses and, 4 weeks later, with EnvA G-Deleted Rabies-eGFP. After 7 d, mice were perfused, and every one in three brain sections was collected (50-µm thickness sections). Starter cells were considered TVA-mCherry and Rabies-eGFP double positive. Upstream Rabies-eGFP cells were then counted using ImageJ software. Owing to proximity with the MeA starter cell location, the lateral hypothalamus (LH), anterior MeA and anterior amygdalar area (AAA) were excluded from the analysis. Brains with more than 70% of starter cells in the MeA were considered for further analysis. Regions with more than 2% of total inputs to MeA^Foxp2^ and MeA^Dbx1^ cells were included in Fig. 6.

### Output axonal projection mapping

To determine the projection patterns of MeA^Foxp2^ and MeA^Dbx1^ cells, every one in three brain sections was collected (50-µm thickness sections) and analyzed in Adobe Photoshop as previously described^55^. In brief, the average pixel intensity of each region of interest (I_raw_) was obtained, and the background intensity from the contralarateral side (l_background_) was substracted (I_signal_) and normalized to the maximum l_signal_ across all brain regions (I_norm_). The average I_norm_ was then calculated for all animals to obtain the average axonal projection intensity for each terminal field. Animals with more than 65% of starter cells in the MeA were considered for analysis. Regions with more than 0.2 normalized intensity were included in Fig. 8. VMHvl was shown in the analysis given its well-established role in territorial aggression^42,58,59^. The LH and anterior MeA were excluded from the analysis owing to their proximity to the starter cells.

### Brain slice electrophysiology

For AOB to MeA circuit mapping experiments, we injected AAV2-Flex-eGFP and AAV9-hSyn-ChrimsonR-tdTomato into the MeA and the AOB, respectively, of Foxp2^cre+/*−*^ male mice or AAV9-hSyn-ChrimsonR-tdTomato into the AOB of Dbx1^cre+/*−*^Ai6^+/*−*^ male mice. Whole-cell patch-clamp recordings were performed on MeA slices from all mice.

Mice were anesthetized with isoflurane, and brains were removed and submerged in ice-cold cutting solution containing (in mM): 110 choline chloride, 25 NaHCO_3_, 2.5 KCl, 7 MgCl_2_, 0.5 CaCl_2_, 1.25 NaH_2_PO_4_, 25 glucose, 11.6 ascorbic acid and 3.1 pyruvic acid. Coronal sections of 275 µm were cut on a Leica VT1200 S vibratome and incubated in artificial cerebral spinal fluid (ACSF) containing (in mM): 125 NaCl, 2.5 KCl, 1.25 NaH_2_PO_4_, 25 NaHCO_3_, 1 MgCl_2_, 2 CaCl_2_ and 11 glucose at 34 °C for 30 min and then transferred to room temperature for cell recovery until the start of recording. Whole-cell voltage-clamp recordings were performed with micropipettes filled with intracellular solution containing (in mM): 135 CsMeSO_3_, 10 HEPES, 1 EGTA, 3.3 QX-314 (chloride salt), 4 Mg-ATP, 0.3 Na-GTP and 8 sodium phosphocreatine (pH 7.3 adjusted with CsOH). Signals were recorded using MultiClamp 700B amplifier and digitized by DigiData1550B with sampling rate of 20 kHz (Molecular Devices). Data were analyzed using Clampfit (Molecular Devices) or MATLAB (Mathworks). To activate ChrimsonR-expressing axons, brief pulses of full-field illumination (pE-300 white; CoolLED, 605 nm, 1-ms duration, 10 repeats, with 6-s interval) were delivered onto the recorded cell. oESPSs and oIPSCs were recorded by holding the membrane potential of recorded neurons at −70 mV and 0 mV, respectively. ACSF, TTX (1 µM), TTX (1 µM) and 4-AP (100 µM) were sequentially used to test if optogenetically evoked responses are monosynaptic. All drugs were pre-applied for 5 min in the slice chamber before data acquisition. Latency was measured as the time difference when the current exceeded 1.5 folds of the standard deviation of baseline compared to the light onset.

### Statistics

All statistical analyses were performed using MATLAB or GraphPad Prism (versions 8 and 9) software. Statistical analyses were two-tailed. Parametric tests, including paired and unpaired *t*-test and one-way ANOVA, were used if distributions passed the Kolmogorov–Smirnov normality test. Normality tests were not performed for one-way ANOVA with missing values, sample size ≤4 and two-way ANOVAs. If data were not normally distributed, non-parametric tests were used. One-sample *t*-test was performed to determine whether the group mean differs from a specific value. When multiple *t*-tests were performed, *P* values were adjusted using the original false discovery rate (FDR) method of the Benjamini–Hochberg process. For comparisons across more than two groups, one-way ANOVA or repeated-measures one-way ANOVA was performed for normally distributed data, followed by Tukey’s multiple comparison tests; Friedman or Kruskal–Wallis tests were used for non-normally distributed data, followed by a two-stage linear step-up procedure of Benjamini, Krieger and Yekutieli multiple comparison tests. Two-way ANOVA was performed for differences between groups with two independent variables, followed by Sidak’s multiple comparison tests. All significant *P* values < 0.05 are indicated in the figures. *P < 0.05; **P < 0.01; ***P < 0.001; ****P < 0.0001. For detailed statistical analysis, see the Source Data associated with each figure.

### Reporting summary

Further information on research design is available in the Nature Portfolio Reporting Summary linked to this article.

## Online content

Any methods, additional references, Nature Portfolio reporting summaries, source data, extended data, supplementary information, acknowledgements, peer review information; details of author contributions and competing interests; and statements of data and code availability are available at 10.1038/s41593-023-01475-5.

### Supplementary information

Reporting Summary

### Source data

Source Data Fig. 1 Statistical Source Data.

Source Data Fig. 2 Statistical Source Data.

Source Data Fig. 3 Statistical Source Data.

Source Data Fig. 4 Statistical Source Data.

Source Data Fig. 5 Statistical Source Data.

Source Data Fig. 6 Statistical Source Data.

Source Data Fig. 7 Statistical Source Data.

Source Data Fig. 8 Statistical Source Data.

Source Data Extended Data Fig. 1 Statistical Source Data.

Source Data Extended Data Fig. 2 Statistical Source Data.

Source Data Extended Data Fig. 3 Statistical Source Data.

Source Data Extended Data Fig. 4 Statistical Source Data.

Source Data Extended Data Fig. 5 Statistical Source Data.

Source Data Extended Data Fig. 6 Statistical Source Data.

Source Data Extended Data Fig. 7 Statistical Source Data.

Source Data Extended Data Fig. 8 Statistical Source Data.

## Figures and Tables

**Extended Data Table 1:**
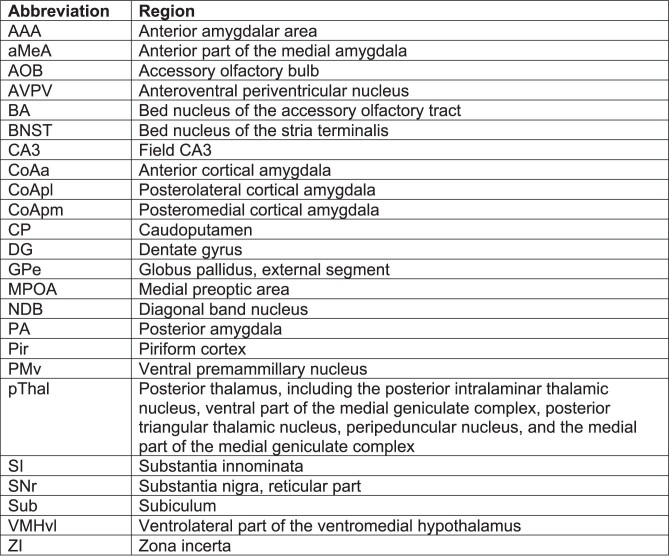
List of brain regions and abbreviations

## Data Availability

Raw values associated with each figure panel can be found in the Source Data files. Fiber photometry recording data, raw representative images and behavior annotations can be downloaded from 10.5281/zenodo.8357846 as of the publication date. Behavior videos, additional histology images and information required to re-analyze the data are available from the corresponding authors upon reasonable request. Source data are provided with this paper.

## Code availability

Custom-written MATLAB code used in the study can be downloaded from 10.5281/zenodo.8357846.
