# Supplementary material for: Transcriptionally defined amygdala subpopulations play distinct roles in innate social behaviors
Source: Nat Neurosci. 2023 Nov 9;26(12):2131–46. doi: 10.1038/s41593-023-01475-5 (PMC10689240; doi:10.1038/s41593-023-01475-5)
Supplement: Supplementary file 1 — Reporting Summary [file 41593_2023_1475_MOESM1_ESM.pdf]

nature portfolio

Corresponding author(s): Julieta E Lischinsky and Dayu Lin.

Last updated by author(s): Sep 15, 2023

Reporting Summary

Nature Portfolio wishes to improve the reproducibility of the work that we publish. This form provides structure for consistency and transparency in reporting. For further information on Nature Portfolio policies, see our Editorial Policies and the Editorial Policy Checklist.

Statistics

For all statistical analyses, confirm that the following items are present in the figure legend, table legend, main text, or Methods section.

n/a	Confirmed	
☐	☒	The exact sample size (*n*) for each experimental group/condition, given as a discrete number and unit of measurement
☐	☒	A statement on whether measurements were taken from distinct samples or whether the same sample was measured repeatedly
☐	☒	The statistical test(s) used AND whether they are one- or two-sided<br>*Only common tests should be described solely by name; describe more complex techniques in the Methods section.*
☐	☒	A description of all covariates tested
☐	☒	A description of any assumptions or corrections, such as tests of normality and adjustment for multiple comparisons
☐	☒	A full description of the statistical parameters including central tendency (e.g. means) or other basic estimates (e.g. regression coefficient) AND variation (e.g. standard deviation) or associated estimates of uncertainty (e.g. confidence intervals)
☐	☒	For null hypothesis testing, the test statistic (e.g. *F*, *t*, *r*) with confidence intervals, effect sizes, degrees of freedom and *P* value noted<br>*Give P values as exact values whenever suitable.*
☒	☐	For Bayesian analysis, information on the choice of priors and Markov chain Monte Carlo settings
☒	☐	For hierarchical and complex designs, identification of the appropriate level for tests and full reporting of outcomes
☒	☐	Estimates of effect sizes (e.g. Cohen's *d*, Pearson's *r*), indicating how they were calculated

Our web collection on statistics for biologists contains articles on many of the points above.

Software and code

Policy information about availability of computer code

Data collection	To collect fiber photometry signals, we used a custom TDT program, OpenEx (Tucker-Davis Technologies). Streampix 5 (Norpix) was utilized for multiple camera recordings. A virtual slide scanner (Olympus, VS120) was used to capture epifluorescent images and a Zen program from LSM 800 (Zeiss) to capture confocal images. Signals from whole-cell voltage-clamp were recorded using a MultiClamp 700B amplifier and digitized by DigiData1550B (Molecular Devices).
Data analysis	For data analysis we used MATLAB software costume code. To manually annotate behaviors we used a custom MATLAB function named 'BehaviorAnnotator' available at: https://github.com/pdollar/toolbox. For tracking of animal movement in the cage, we used a custom-written Matlab GUI and code available at: https://github.com/pdollar/toolbox. For statistical analysis we used the MATLAB software and GraphPad Prism versions 8 and 9 software. Epifluorescent and confocal imaging data were analyzed by ImageJ1.52N and Adobe Photoshop 2020 and 2023 softwares using their respective counting tools, except for counting DAPI were we used the 'analyze particles' feature and manually corrected. Whole-cell patch clamp data were analyzed using Clampfit (Molecular Devices) or MATLAB. Custom codes are available from the corresponding authors upon reasonable request.

For manuscripts utilizing custom algorithms or software that are central to the research but not yet described in published literature, software must be made available to editors and reviewers. We strongly encourage code deposition in a community repository (e.g. GitHub). See the Nature Portfolio guidelines for submitting code & software for further information.

Data

Policy information about availability of data

All manuscripts must include a data availability statement. This statement should provide the following information, where applicable:

- Accession codes, unique identifiers, or web links for publicly available datasets
- A description of any restrictions on data availability
- For clinical datasets or third party data, please ensure that the statement adheres to our policy

The datasets supporting the findings of this study have been deposited to Zenodo. Any additional information required to reanalyze the data is available upon reasonable request from the corresponding authors.

Human research participants

Policy information about studies involving human research participants and Sex and Gender in Research.

Reporting on sex and gender	N/A
Population characteristics	N/A
Recruitment	N/A
Ethics oversight	N/A

Note that full information on the approval of the study protocol must also be provided in the manuscript.

Field-specific reporting

Please select the one below that is the best fit for your research. If you are not sure, read the appropriate sections before making your selection.

☒ Life sciences ☐ Behavioural & social sciences ☐ Ecological, evolutionary & environmental sciences

For a reference copy of the document with all sections, see nature.com/documents/nr-reporting-summary-flat.pdf

Life sciences study design

All studies must disclose on these points even when the disclosure is negative.

Sample size	Sample sizes in the data are similar to those in previous work in the field (Hashikawa et al., Nat. Neurosci., 2017; Yamaguchi et al., Nat Neurosci., 2020). No statistical method was used to pre-determine sample sizes.
Data exclusions	For fiber photometry experiments, animals that did not show correct unilateral viral infection and fiber placement were removed from the analysis. For chemogenetic experiments, animals that did not show correct bilateral viral infection were removed from analysis. For monosynaptic-retrograde rabies input mapping, animals with less than 70% of starter cells in MeA were pre-established to be excluded from analysis. Regions with more than 2% of total inputs to either the posterior MeA Foxp2+ or posterior MeA Dbx1-derived cells were pre-established to be included in the study. Due to close proximity with the posterior MeA starter cells, the LH, anterior MeA and AAA were pre-established to be excluded from analysis. For output axonal projection mapping, animals with less than 65% of starter cells in the posterior MeA were excluded from analysis. Regions with more than 0.2 normalized intensity were pre-established to be included in the study. It was pre-established that the LH and anterior MeA regions were excluded from analysis due to close proximity to the posterior MeA starter cells. For whole-cell voltage-clamp recordings, we excluded recorded cells that were located in the anterior MeA.
Replication	Experimental findings were reliably reproduced among all subjects in all experiments comprised of multiple cohorts. Detailed number of cohorts included for each experiment is highlighted in the Statistic Table.
Randomization	For fiber photometry experiments, experimental mice were selected randomly and social stimuli were presented in a pseudo-random fashion. For chemogenetic experiments, experimental and control animals were randomly selected from naive mice with no prior sexual or aggressive experience. For chemogenetic silencing experiments, naive mice randomly selected were then trained (by repeated resident intruder tests) for aggressive behaviors. For retrograde, anterograde and histology experiments, experimental animals were selected randomly.
Blinding	In this study, experiments were not performed blindly as the experimental conditions (control vs experimental groups) were clear to the experimenters and the analysis were carried out by using a recording system, which was not subjective to human bias. During the behavioral annotations, the experimenter was blind to the GCaMP6 signal or to the behavioral response.

Reporting for specific materials, systems and methods

We require information from authors about some types of materials, experimental systems and methods used in many studies. Here, indicate whether each material, system or method listed is relevant to your study. If you are not sure if a list item applies to your research, read the appropriate section before selecting a response.

Materials & experimental systems

n/a	Involved in the study
☐	☒ Antibodies
☒	☐ Eukaryotic cell lines
☒	☐ Palaeontology and archaeology
☐	☒ Animals and other organisms
☒	☐ Clinical data
☒	☐ Dual use research of concern

Methods

n/a	Involved in the study
☒	☐ ChIP-seq
☒	☐ Flow cytometry
☒	☐ MRI-based neuroimaging

Antibodies

Antibodies used	We list all antibodies used with their concentration and catalogue number in the Methods section in the 'Immunohistochemistry and imaging analysis' subheading. Primary antibodies used were rabbit anti-718 Foxp2 (1:500, abcam ab16046), rat anti-GFP (1:1000, Nacalai 04404-84), and rabbit anti-mCherry (1:1000, TaKaRa Living Colors DsRed Polyclonal Ab 632496). Secondary antisera used were donkey anti-rat Alexa 488 (1:300; Jackson ImmunoResearch 712-545-150), and donkey anti-rabbit Cy3 (1:1000, Jackson ImmunoResearch 711-165-152).
Validation	https://www.abcam.com/foxp2-antibody-ab16046.html<br>https://www.nacalaiusa.com/products/view/101/anti-gfp-rat-igg2a-monoclonal-gf090r<br>https://www.takarabio.com/products/antibodies-and-elisa/fluorescent-protein-antibodies/red-fluorescent-protein-antibodies<br>https://www.jacksonimmuno.com/catalog/products/712-545-150<br>https://www.jacksonimmuno.com/catalog/products/711-165-152

Animals and other research organisms

Policy information about studies involving animals; ARRIVE guidelines recommended for reporting animal research, and Sex and Gender in Research

Laboratory animals	The mice used in this study are described in the Methods section in the 'Mice' subheading. Experimental and stimulus mice were housed under a 12 hr light-dark cycle (10a.m. to 10p.m. dark) with water and food ad libitum. Holding and experimental room temperatures were maintained at 20-22oC and humidity was maintained between 30-70%, with an average humidity of ~45%. After surgical procedures, all experimental animals were single-housed. The Foxp2cre mice were originally provided by Dr. Richard Palmiter (now Jackson stock no. 030541). The Dbx1cre mice were originally provided by Dr. Alessandra Pierani and crossed to the Flp excised and Cre-inducible LSL-FlpO mouse line or to the Ai6 mouse line (Jackson stock no. 028584 and no. 007906 respectively). Both Foxp2cre and Dbx1cre mice are black, while the fur color of LSL-FlpO mice is agouti. Stimulus animals were C57BL/6N and 129S4/SvJae group-housed females, pups (P1-P7) and group-housed BALB/c males purchased from Charles River and bred in-house. Adult mice were between 2 to 8 months of age. Juvenile experimental mice were P11 when undergoing surgery for GCaMP6 experiments.
Wild animals	This study did not involve wild animals.
Reporting on sex	This study used only male mice.
Field-collected samples	This study did not involve samples collected from the field.
Ethics oversight	All animal procedures were approved by the Institutional Animal Care and Use Committee (IACUC) of NYU Langone Health. We complied with all ethical regulations.

Note that full information on the approval of the study protocol must also be provided in the manuscript.
